# Occurrence of antibiotics and bacterial resistance genes in wastewater: resistance mechanisms and antimicrobial resistance control approaches

**DOI:** 10.1007/s11274-022-03334-0

**Published:** 2022-07-04

**Authors:** Christopher Mutuku, Zoltan Gazdag, Szilvia Melegh

**Affiliations:** 1 Department of General and Environmental Microbiology, Faculty of Sciences, University of Pécs, Ifjúság u. 6, Pecs, 7624 Hungary; 2 Department of Medical Microbiology and Immunology, Medical School, University of Pécs, 7622 Pecs, Hungary

**Keywords:** Antibiotics, Bacteria, Resistance genes, Wastewater

## Abstract

Antimicrobial pharmaceuticals are classified as emergent micropollutants of concern, implying that even at low concentrations, long-term exposure to the environment can have significant eco-toxicological effects. There is a lack of a standardized regulatory framework governing the permissible antibiotic content for monitoring environmental water quality standards. Therefore, indiscriminate discharge of antimicrobials at potentially active concentrations into urban wastewater treatment facilities is rampant. Antimicrobials may exert selective pressure on bacteria, leading to resistance development and eventual health consequences. The emergence of clinically important multiple antibiotic-resistant bacteria in untreated hospital effluents and wastewater treatment plants (WWTPs) has been linked to the continuous exposure of bacteria to antimicrobials. The levels of environmental exposure to antibiotics and their correlation to the evolution and spread of resistant bacteria need to be elucidated to help in the formulation of mitigation measures. This review explores frequently detected antimicrobials in wastewater and gives a comprehensive coverage of bacterial resistance mechanisms to different antibiotic classes through the expression of a wide variety of antibiotic resistance genes either inherent and/or exchanged among bacteria or acquired from the reservoir of antibiotic resistance genes (ARGs) in wastewater systems. To complement the removal of antibiotics and ARGs from WWTPs, upscaling the implementation of prospective interventions such as vaccines, phage therapy, and natural compounds as alternatives to widespread antibiotic use provides a multifaceted approach to minimize the spread of antimicrobial resistance.

## Introduction

One of the major milestones of the last century was the advent of antimicrobial pharmaceuticals, which are currently widely applied in human and veterinary medicine to prevent and manage infections, and in animal husbandry as growth promoters (Cycoń et al. 2019). Antibiotics are a class of active pharmaceutical compounds that are widely consumed around the world to inhibit bacterial proliferation through cell destruction or growth inhibition (Kümmerer 2009). Data from scientific literature, national and regional surveillance systems from numerous countries over time indicates a steadily increasing antibiotic use worldwide (30%), primarily due to rising demand in low and middle-income countries (Gelband et al. 2015). This unprecedented increase in antibiotic use continues to raise concern about their potentially harmful effects on the environment. (Bengtsson et al. 2018). However, despite their potential environmental and health effects, the use of these agents has revolutionized health care by improving hygiene and considerably changing the outcome of bacterial infections, which has in turn, significantly increased the average expected lifespan (Carvalho and Santos 2016; Chowdhury et al. 2017). Their consumption varies from region to region and from country to country (Göbel et al. 2005). Studies have shown that many of these antimicrobials are not completely metabolized during therapeutic use and an estimated 30–90% end up being excreted as active substances into sewage water, resulting in the presence of multiple classes of antibiotics being widely detected in various urban wastewater treatment plants and the receiving environment around the world (Chen et al. 2006; Li 2014). Their consumption patterns influence the extent of their environmental contamination where an increase in consumption, especially during the cold season when the frequency of infections is higher, elevates their occurrence in environmental systems (Wang et al. 2020), which correlates with the emergence of multiresistant bacteria and their rapid expansion (Levy 2002). Due to their widespread application, antimicrobials have been and continue to be discharged into the environment via wastewater of human origin from different sources, including households (domestic), hospitals (clinical), veterinary and animal husbandry, and pharmaceutical factories (industrial) (Kemper 2008). Following their discharge into water systems, several antimicrobials and their by-products are detected in the environment at concentrations that range from ng L^−1^ to µg L^−1^ (Seifrtova et al. 2009). They reach the aquatic environment mainly through the flow of wastewater treatment plant effluent into surface water or into groundwater (Carvalho and Santos 2016). They are considered emergent micropollutants with the potential to create selective pressure for the development of microbial resistance in the environment (Kümmerer 2009; Kumar et al. 2019). The permissible limits of the widely used substances of priority concern which may pose potential risks in aqueous media, excluding antibiotics, were set out by the EU Directive 2013/39/EU within the European Union to maintain environmental quality standards and ecological integrity (Ricci et al. 2016). Upon examining various ecotoxicological reports, the multiple threats posed by antibiotics as environmental contaminants were, however, recognized and the EU, alongside other countries, introduced a regulatory framework to monitor emerging substances of concern in the aquatic environment (Wang et al. 2020). For instance, in its decision of 2015 (EU Decision, 2015/495 of March 20, 2015), the EU Commission established a watch list of three antibiotics belonging to the macrolide class, namely clarithromycin, azithromycin, and erythromycin as contaminants of priority concern due to their potential risk to the aquatic environment, and thereafter added amoxicillin and ciprofloxacin to the watch list in 2018 (EU Decision, 2018/840 of June 5, 2018) (Felis et al. 2020). The occurrence of antimicrobial compounds in the environment varies among the different antimicrobial classes depending on their frequency of usage, and a major concern about their presence in the environment relates to the emergence of antibiotic resistance genes (ARGs) and the evolution of antibiotic resistant bacteria (ARB), which endanger pharmaceuticals’ ability to control microbial pathogens (Kumar et al. 2019). Continuous antimicrobials exposure has seen more antibiotics become less effective due to the growing resistance observed among the primary and opportunistic pathogens, resulting in higher medical and economic costs and increased mortality (Zhen et al. 2019). Antimicrobials are frequently administered in health care facilities. However, hospital effluents are not the primary source of resistant bacteria in the environment since they contribute less than 2% of the total volume of wastewater and, therefore, other sources require monitoring (Carraro et al. 2016). Human excreta-containing habitats such as wastewater treatment plants (WWTPs) and compost toilets, together with animal farms and aquaculture, are thought to be reservoirs for the emergence and propagation of resistant bacteria (Korzeniewska and Harnisz 2018; Zhou et al. 2018; Karkman et al. 2019). Hospital effluents eventually enter into WWTPs, which are characterized by the abundance of organic and inorganic nutrients and the proximity of cells, enhancing individual cell-to-cell interactions. The presence of antimicrobial residues and other suitable conditions such as temperature and pH make WWTPs ideal for ARB development and may promote the proliferation of ARB and the eventual spread of antibiotic resistance genes (ARGs) (Berendonk et al. 2015; Krzeminski et al. 2019). Furthermore, the antibiotic resistance patterns described in clinical settings appear to correlate with those observed in WWTPs (Pärnänen et al. 2019).

Vast studies have reported the widespread presence of antibiotics, especially in WWTPs over time. However, many of these studies do not compare the situation in a wide geographical region, and those conducted within particular countries explore a few compounds found in a small number of WWTPs within that country (Rodriguez-Mozaz et al. 2020). Therefore, there is a significant gap for implementing mitigation measures. In addition, the data reported in different studies from country to country are not sufficiently comparable due to a lack of standardized methodologies. Thus, it is a challenge to develop environmental protection guidelines that can be applied universally. To allow for the evaluation of vast trends in antibiotic occurrence, it would be important to conduct monitoring studies in a wide region that covers many WWTPs and a variety of compounds using standardized protocols. Monitoring antibiotic contamination is important, more so given its link to antibiotic resistance (AR), which is a global public health concern (Hendriksen et al. 2019). Combating antibiotic resistance requires being addressed in a context that integrates environmental and human health concerns, with a focus on antibiotic environmental contamination that provides a broader perspective with diverse empirical data on the correlation between antibiotics in the environment and the evolution of antibiotic resistant bacteria, which requires further investigation. The One Health initiative’s perspective, championed by the World Health Organization (WHO), envisions human health issues including AR in the context of humans, animals, and the environment (WHO 2020). Similarly, the United Nations Sustainable Development Goals (UNSDGs), are aimed at promoting sustainable improvement in the health of millions of people by conquering contamination of surface water, groundwater, and wastewater treatment plants (WWTP) (Zhou et al. 2020). Understanding how ARB and ARGs spread from WWTPs and their role in resistance dissemination is critical for developing mitigation measures to limit the spread of AR in the environment. Other reviews have covered the major chemical groups of antibiotics commonly detected in wastewater systems in greater detail (Pazda et al. 2019; Felis et al. 2020; Uluseker et al. 2021), hence this paper discusses them briefly. The paper gives an insight into the role of antibiotics in promoting the evolution and development of resistance in antimicrobial contaminated environments. Some major bacterial resistance mechanisms to the key antibiotic groups (with associated ARGs) where antibiotics, bacteria and ARGs, occur in the same wastewater environment are elucidated, a perspective which appears to have been overlooked in other reviews (Blair et al. 2015; Pazda et al. 2019; Felis et al. 2020). It also gives an account of some case studies that have demonstrated a possible correlation between environmental antimicrobial contamination and antibiotic resistance. The promising antibiotic alternative approaches that have demonstrated prospects in combating the current state of antibiotic resistance, which complement the technologies applied in the removal of antibiotics and ARGs from wastewater treatment plants, are briefly outlined.

## Antibiotics classes frequently detected in aqueous environments

Pharmaceuticals are widely used in livestock production and in agriculture, in addition to human use (Koch et al. 2021). Each year, approximately 24.6 million pounds of antibiotics are used in livestock farming (Van et al. 2020). This has become a global practice because low-dose antibiotics were found to boost animal and bird growth by adding them to animal feeds (Kumar et al. 2018). Their extensive use in animal production forms the main source of environmental antibiotics (Kinney and Heuvel 2020). Numerous studies have reported the presence of pharmaceutical compounds or their metabolites in the geosphere and biosphere (Bartrons and Penuelas 2017; Riaz et al. 2018), with pharmaceutical contaminants being reported in polar regions, the most pristine environment on earth (González-Alonso et al. 2017). Some case studies of compounds documented in the European WWTPs and hospital effluents as well as those from a few other regions are cited for the purpose of this discussion. The recent data on variation in the consumption rate of antibacterial agents within the European Union/European Economic Area in both the hospital and community sectors in a two year period is presented in Table 1. This gives an insight into the frequency of antimicrobial usage based on the commonly prescribed chemical classes.

**Table 1:** Average consumption of antibacterials for systemic use in the community and hospital sector in the European Union/ European Economic Area, in 2019 and 2020 (expressed as DDD per 1000 inhabitants per day (ECDC 2020)

Antimicrobial compound	Community sector		Hospital sector	
	2019	2020	2019	2020
Tetracyclines	2.1	1.6	0.09	0.05
β-Lactams (penicillins)	8	6.5	0.65	0.48
Other β-lactam antibacterials	2	1.7	0.4	0.43
Sulfonamides and trimethoprim	0.6	0.5	0.07	0.07
Macrolides, lincosamides and streptogramins	2.8	2.4	0.16	0.17
Quinolones	1.3	1.2	0.17	0.16
Other antibacterials	1.1	1	0.17	0.16
Other groups	0.1	0.1	0.06	0.05

Other groups are amphenicols, aminoglycosides and combination of antibacterials

It has been observed that the concentrations of antibiotic classes vary based on the antimicrobial compound and environmental matrix and the load tends to decrease from wastewater generated by human activity to the surface and groundwater (Carvalho and Santos 2016). The pattern and consumption rate, excretion, and the efficacy of elimination by wastewater treatment processes, together with weather conditions, especially rainwater, usually influence the concentrations of the antibiotics detected in wastewater treatment plant influents and effluents (Osorio et al. 2012). Certain antimicrobial agents, especially macrolides, sulfonamides, quinolones, and trimethoprim, persist in the aqueous environment and are among the most frequently detected substances in the environmental matrices due to their stability and because they are frequently prescribed in veterinary and human medicine (Wang and Wang 2016; Korzeniewska and Harnisz 2020). Considering their occurrence as demonstrated in various studies, the WWTP effluents containing high concentrations of these antibiotics are discharged into surface water, especially rivers, which subsequently become the main outlets of such antimicrobials into the rest of the natural aqueous environment. Some of the antimicrobial classes detected in the aqueous environments are described briefly.

### β-Lactams

This group consists of a class of broad-spectrum antimicrobial compounds, which are the most frequently administered antimicrobials in all European countries similar to the rest of the world (Korzeniewska and Harnisz 2020). β-Lactams are structurally characterized by a β-lactam ring which is highly susceptible to hydrolysis by a variety of reagents, both biotic (enzymatic and biological degradation) and abiotic (chemical degradation) processes. The β-lactam ring is easily destroyed by extremes in pH, light, heat, solvents like water and methanol (Deshpande et al. 2004). The variation of β-lactams occurrence in the environment during the year depends on therapeutic usage and consumption patterns. They rarely persist in the environment due to their unstable property in spite of being widely consumed. However, the β-lactams, penicillin G and V were mostly found in raw wastewater samples, whereas amoxicillin, a synthetic derivative of penicillin, and cefuroxime, a second-generation cephalosporin, are much more stable and are frequently found in hospital effluents as well as raw wastewater (Michael et al. 2013; Harrabi et al. 2018).

### Aminoglycosides

The usage of aminoglycosides in clinical practice is often restricted due to their adverse effects and toxic potential, which makes their contamination of the aqueous environment mostly associated with their application in veterinary medicine. Despite their low consumption, aminoglycosides have been detected in raw and treated wastewater, which was attributed to effluents from hospitals and wastewater from factories producing these pharmaceuticals (Tahrani et al. 2016). Several aminoglycosides were detected in wastewater treatment plant influents and effluents in various ranges, including kanamycin B, sisomicin, gentamicin, and neomycin (Tahrani et al. 2016). In Poland, the occurrence of aminoglycosides neomycin, streptomycin, and dihydrostreptomycin investigated in water samples drawn from supply systems in different animal farms yielded only neomycin (Gbylik-Sikorska et al. 2015).

### Quinolones and fluoroquinolones

The quinolone class of chemically synthesized antibiotics was among the latest to be introduced in clinical practice. They are frequently used and their consumption in human medicine is estimated to account for 7% of the total antimicrobial consumption (Szymańska et al. 2019). Fluoroquinolones are mobile in the aquatic environment due to their hydrophilic property, which explains their presence in both groundwater and drinking water samples (Hanna et al. 2018; Reis et al. 2019). It is this ability to rapidly spread in the environment that necessitated the inclusion of ciprofloxacin in the watch list of the EU commission, Decision of 2018. Their occurrence in different aqueous environmental matrices has been reported, with the maximum concentrations typically occurring in hospital effluents and WWTP influents. Ciprofloxacin and ofloxacin appear to be the dominant ones detected in wastewater with high detection frequency and high concentration (Lindberg et al. 2007). Several other quinolones and fluoroquinolones, which include pipemidic acid, nalidixic acid, moxifloxacin, and gatifloxacin, have been detected in WWTPs (Zhang and Li 2011) (1). European WWTP influents and effluents have reported quinolones in various concentrations (Santos et al. 2013). Ciprofloxacin, for example, has been found in hospital effluent from Spain, Sweden, Portugal, and Italy at concentrations of tens of µg L^−1^ (Lindberg et al. 2004; Gracia-Lor et al. 2012; Verlicchi et al. 2012; Gros et al. 2013; Santos et al. 2013), which presents hospital effluents as important input sources of quinolones into wastewater.

### Sulfonamides and diaminopyrimidine

Sulfamethoxazole is the representative drug among the sulfonamides and is currently the most frequently used drug in this class, making the compound one of the most common substances found in the environment (Hanna et al. 2018; Loos et al. 2018). Studies have shown that sulfonamides are partially excreted unchanged, primarily through urine (Prescott 2013). Their occurrence in different aqueous environmental matrices in various regions over the last decades has been documented. The concentration of sulfonamides in WWTP influents and effluents was found to range from tens to hundreds of ng L^−1^, and this is attributed to their consumption in the community sector (Golovko et al. 2014; Papageorgiou et al. 2016). Sulfamethoxazole, the most common sulfonamide, has been found in WWTP influents and effluents in Germany, Portugal and Kenya (Santos et al. 2013; Rossmann et al. 2014; Ngumba et al. 2016). Very high concentrations of sulfonamides (20 × 10^3^ ng ml^−1^) have been detected in pig farm wastewater, and the detection of sulfamethazine, for example, has been suggested to serve as a marker for livestock source contamination in Vietnam (Managaki et al. 2007). Trimethoprim is the representative diaminopyrimidine that is used in combination with sulfonamides to increase the bactericidal effect achieved through synergy. A combination of trimethoprim and the sulfonamide, sulfamethoxazole (Co-trimoxazole), has widespread use in both human and veterinary medicine. Trimethoprim has been determined in WWTPs and hospital effluents in the UK, Croatia, Greece, Italy, and Sweden (Kasprzyk-Hordern et al. 2009; Verlicchi et al. 2012; Santos et al. 2013; Kosma et al. 2014; Mendoza et al. 2015).

### Tetracyclines

Tetracyclines comprise both natural antibiotics such as tetracycline, chlortetracycline, oxytetracycline, and semi-synthetic drugs such as doxycycline and demeclocycline. Tetracycline is a broad-spectrum antibiotic that has been widely used to prevent infections in humans and animals, and as a growth promoter in animal feeding at sub-therapeutic dose levels (Sabino et al. 2019). Tetracycline is widely distributed in animal farms, and in the gut of migratory birds, and has potential side effects on human health (Cao et al. 2020). Although they are less frequently used in human medicine, they have been identified in samples of wastewater, surface water, and drinking water (Azanu et al. 2018b; Hanna et al. 2018). Humans and animals excrete over 70% of tetracycline antibiotics in an active form to the environment and, owing to their highly hydrophobic property and low volatility, tetracyclines are very stable in the aquatic environment and are commonly detected in WWTPs (Daghrir and Drogui 2013). They form stable complexes with cations which makes them more likely to bind to suspended matter or sewage sludge during wastewater treatment (Collado et al. 2014). Tetracycline is the most common substance detected in WWTPs (Opriş et al. 2013; Vergeynst et al. 2015). Five tetracycline antibiotics, including doxycycline, tetracycline, oxytetracycline, and chlortetracycline were found in hospital samples and WWTPs influent and effluent in Sweden, Hong Kong, Norway, and Germany (Yang et al. 2005; Lindberg et al. 2006; Minh et al. 2009; Watkinson et al. 2009; Rossmann et al. 2014).

### Macrolides

Macrolide antibiotics are a critical class of compounds due to their significant consumption in hospitals and they enter into wastewater as unchanged parent compounds upon excretion via bile and feces after being hardly metabolized in the body and the continuous application in veterinary and human medicine has contributed to the presence of these antibiotics in aqueous matrices due to their stability (Nnadozie et al. 2017). These compounds are prevalent in the natural environment, especially WWTPs, where the quantities of the macrolides, tylosin, roxithromycin, azithromycin, and clarithromycin have been determined in raw sewage and the treated effluent (Yang and Carlson 2004; Göbel et al. 2005; Petrovic et al. 2006; Spongberg and Witter 2008; Lin et al. 2009; Watkinson et al. 2009). Some macrolides, particularly clarithromycin and azithromycin are among the most commonly detected antimicrobials (Verlicchi et al. 2012; Loos et al. 2018). Wastewater effluents form key input sources of macrolides into rivers as evidenced by their presence in rivers in Spain and France (Valcarcel et al. 2011; Moreno-González et al. 2014). Although erythromycin is the parent antibiotic, a high concentration of its metabolite dehydrated erythromycin-H_2_O has been found in both raw sewage and treated wastewater effluent (Kasprzyk-Hordern et al. 2009; Minh et al. 2009). The parent antibiotic, erythromycin was found in both the influent and effluent (Ternes et al. 2007). Table 2 shows residues of the various representative substances of the antibiotic classes that have been detected at various concentrations in raw wastewater (including hospital effluents) and treated wastewater.

**Table 2:** Occurrence of antimicrobial compounds in raw wastewater (hospital effluents and WWTP influent) and treated wastewater in ng/L, partly adapted from (Felis et al. 2020)

Class/compound	Raw wastewater	Treated wastewater
*β-Lactams* Penicillin G	18–6196 (Loos et al. 2013, 2018; Ruff et al. 2015), 13800 (Watkinson et al. 2009)	47–1205 (Loos et al. 2013, 2018; Ruff et al. 2015), 2000 (Watkinson et al. 2009)
Penicillin V	nd-160 (Gros et al. 2013; Michael et al. 2013)	
Amoxicillin	33800 (Azanu et al. 2018b), 2.0–57, hospital effluent (Azanu et al. 2018b; Thai et al. 2018)	nd-116400 (Gros et al. 2013; Azanu et al. 2018b)
Cefotaxime	1100 (Watkinson et al. 2009)	< 15 (Watkinson et al. 2009)
Cefuroxime	49–24380 (Ribeiro et al. 2018), 246, hospital effluent (Thai et al. 2018)	7860 pharma factory (Thai et al. 2018)
*Aminoglycosides* Kanamycin B	500–7500 (Tahrani et al. 2016)	700–5400 (Tahrani et al. 2016)
Sisomicin	2300–6700 (Tahrani et al. 2016)	1000–3900 (Tahrani et al. 2016)
Gentamicin	500–1600 (Tahrani et al. 2016), 400–7600 (Löffler and Ternes 2003)	200–600 (Tahrani et al. 2016)
Neomycin	1800–16400 (Tahrani et al. 2016)	400–11200 (Tahrani et al. 2016)
Amikacin	2300 (Tahrani et al. 2016)	1000 (Tahrani et al. 2016)
Streptomycin	2700 (Tahrani et al. 2016)	1200 (Tahrani et al. 2016)
*Fluoroquinolones* Ciprofloxacin	3700 (Verlicchi et al. 2012), 34500 (Matongo et al. 2015), 3600–101000, hospital effluent (Lindberg et al. 2004) 1400–26000, hospital effluent (Verlicchi et al. 2012)	1100 (Verlicchi et al. 2012)
Levofloxacin		4–836 (Rossmann et al. 2014)
Ofloxacin	11.1–1330 (Birošová et al. 2014; Dong et al. 2016), 23–510 hospital effluent (Verlicchi et al. 2012)	0.3–527 (Golovko et al. 2014; Dong et al. 2016)
Norfloxacin	<LOQ-5411 (Dong et al. 2016; Östman et al. 2017), 450–2200, hospital effluent (Verlicchi et al. 2012)	0.2–628 (He and Blaney 2015; Dong et al. 2016)
*Sulfonamides* Sulfamethoxazole	6500, 8700, 13000, 2000, 54800 (Lindberg et al. 2004; Verlicchi et al. 2012; Santos et al. 2013; Ngumba et al. 2016)	3340 (Ngumba et al. 2016)
Sulfapyrydyne	60–230 (Göbel et al. 2005)	0.4–230 (Göbel et al. 2005)
Sulfamethazine	4010 (Li and Zhang 2011)	
*Trimethoprim*	1500–6000 (Verlicchi et al. 2012; Kosma et al. 2014), 4250–72900 (Ngumba et al. 2016), 100–4300 (Göbel et al. 2005; Watkinson et al. 2009; Li and Zhang 2011), < 3000, hospital effluent (Santos et al. 2013)	60–3000 70, 65–800 (Göbel et al. 2005; Li and Zhang 2011; Loos et al. 2018)
*Tetracyclines* Tetracycline	58–1960 (Azanu et al. 2018b; Lorenzo et al. 2018), 13–1598, hospital effluent (Azanu et al. 2018b; Lorenzo et al. 2018; Wang et al. 2018a)	1400–146000 (Opriş et al. 2013; Vergeynst et al. 2015)
Doxycycline	1.8–264 (Azanu et al. 2018b; Hanna et al. 2018), 24–120, hospital effluent (Azanu et al. 2018b) 75–1487, hospital effluent (Azanu et al. 2018b; Wang et al. 2018a)	2210 (Lindberg et al. 2006), 1420(Minh et al. 2009), 14–49(Azanu et al. 2018b)
Oxytetracycline	350 (Watkinson et al. 2009), 43–233 (Azanu et al. 2018a), 24–120 hospital effluent (Azanu et al. 2018b)	250 (Watkinson et al. 2009), 2.4–24(Azanu et al. 2018a)
Chlortetracycline	270 (Yang et al. 2005)	
*Macrolides* Erythromycin	830 (Ternes et al. 2007), 1100 (Matongo et al. 2015), 9–294 (Tylová et al. 2013)	620 (Ternes et al. 2007), 160 (Matongo et al. 2015), 886 (Gracia-Lor et al. 2012; Tylová et al. 2013)
Tylosin	1150 (Yang and Carlson 2004), 55–180 (Watkinson et al. 2007)	3400 (Watkinson et al. 2009)
Roxithromycin	810 (Göbel et al. 2005)	540 (Göbel et al. 2005)
Azithromycin	450 (Petrovic et al. 2006), 1083 (Lara-Martín et al. 2014)	400 (Göbel et al. 2005), 0–380 (Al Aukidy et al. 2012; Lara-Martín et al. 2014)
Clarithromycin	1433 (Lin et al. 2009), 122 (Watkinson et al. 2009; Lara-Martín et al. 2014)	996 (Spongberg and Witter 2008), 8–460 (Al Aukidy et al. 2012; Gracia-Lor et al. 2012; Lara-Martín et al. 2014)

*nd* not detected, *LOQ* limit of quantification

## Dissemination routes of antimicrobial pharmaceuticals and ARGs in the environment

**Fig. 1:**
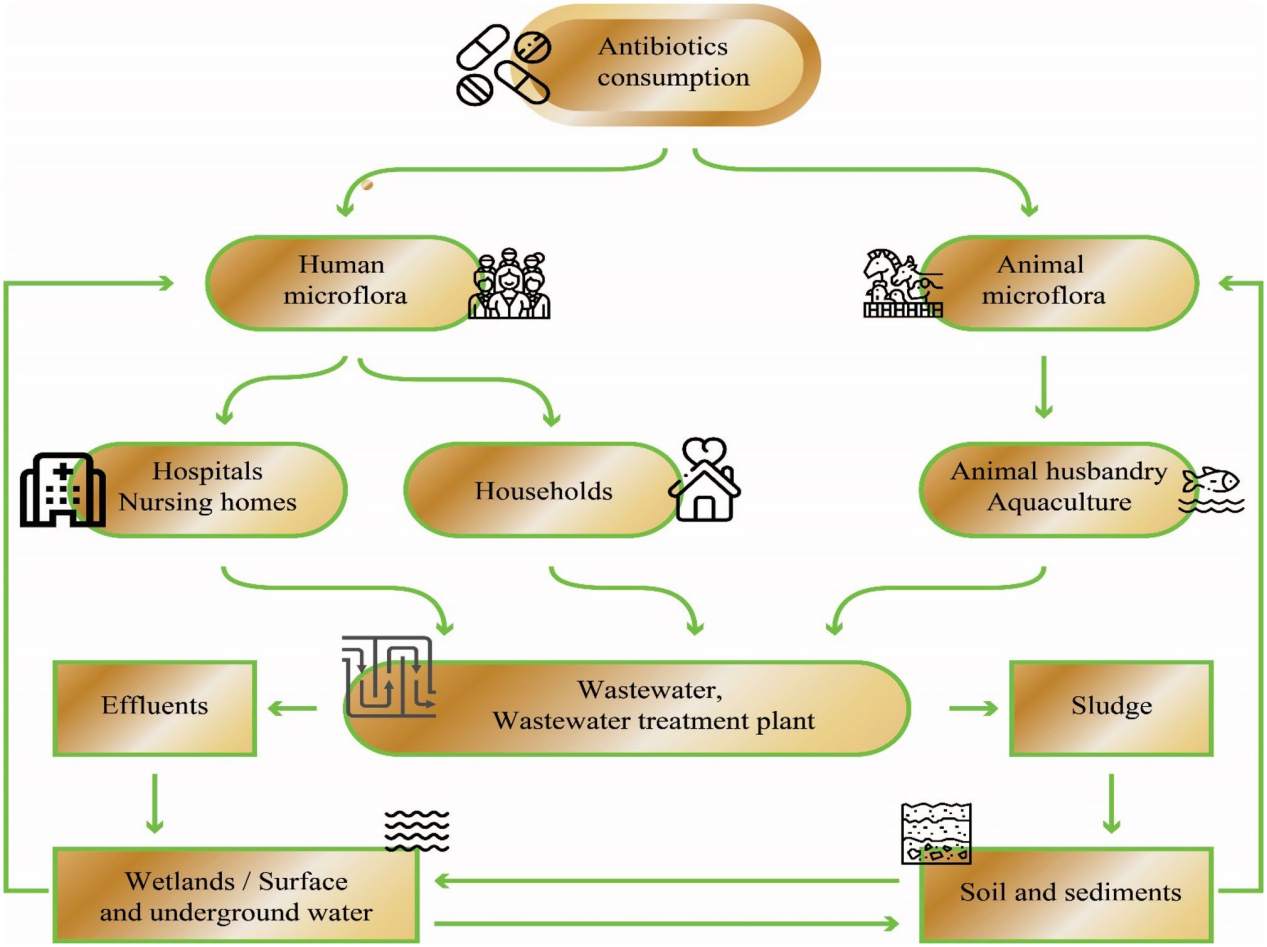
Antibiotics and bacteria from the human population, veterinary medicine, and food-producing animals taking antibiotics enter various habitats such as soil and surface water via excreta, through effluents, and biosolids from wastewater treatment plants. Antibiotics, ARGs, and resident environmental bacteria mix in the various compartments, spurring the emergence and spread of ARB and ARGs in the bacterial community, and they can eventually end up in animal hosts, including humans

Antibiotic overuse, inappropriate prescription, and extensive use of antibiotics in agriculture are linked to the widespread occurrence of antibiotics in the environment (Chowdhury et al. 2017). These and other anthropogenic activities that result in the discharge of wastewater containing antibiotics and/or their metabolites into environmental matrices have been attributed to the increasing antimicrobial resistance due to the rapid evolution of bacteria facilitated by the acquisition of resistance from the reservoir of ARGs, which has a direct impact on the control of microbial pathogens in humans and animals (Kemper 2008; Zhang et al. 2009a). Aquatic environments, especially WWTPs, serve as sinks for massive loads of pharmaceutical compounds, including personal care products and antibiotics, and provide optimal conditions where antibiotic resistant bacteria develop and proliferate and ARGs spread (Kim et al. 2007). Dissemination of antibiotics and ARGs occurs in habitats that provide ideal environments for their spread and circulation between humans, animals, and the external environment. Figure 1 depicts several habitats that are ideal for recombination events and subsequent genetic exchange where the future evolution of resistance among microbes in the environment occurs. Human and animal microflora consisting of diverse bacterial species form the primary habitat in which antibiotics assigned for prevention or therapy exert their actions. Animal and human digestive systems provide suitable residence for bacteria along with sub-lethal doses of antibiotics, which might be potential niches for the propagation of antibiotic resistance (Chopra and Roberts 2001). Environments where susceptible individuals are often overcrowded with possible exposure to bacterial genetic exchange, such as hospitals, nursing/retirement homes (which serve as long-term care facilities), and animal farms, constitute the secondary habitat. Antibiotics and other antimicrobial residues in wastewater that originate from secondary residences find their way into WWTPs where they mingle with bacteria. The wastewater treatment facilities, which constitute the tertiary habitat, provide suitable conditions for mixing and genetic exchange (Berendonk et al. 2015). Soil or sediments and surface or groundwater environments provide the final habitat in which bacteria originating from previous habitats continuously mix and interact with the broader microbial communities in the environment. The interconnection among these habitats creates a niche that breeds resistant bacteria and ARGS, which circulate in the ecosystem and may eventually be re-introduced into human and animal environments. The strategies employed by humans to regulate the introduction of active antimicrobial agents and bacteria into these sites, such as pre-treatment of hospital effluents and enhancing antibiotic stewardship programs, minimize the possibility of the microbes evolving antibiotic resistance.

## Antibiotics biodegradation mechanisms and pathways

The numerous processes involved in the removal of contaminants in WWTPs have been documented. For example, sorption onto biological sludge in biological wastewater treatment systems plays a significant role in antibiotics’ removal from the aqueous phase. However, antibiotics such as sulfonamides (e.g., sulfamethoxazole-SMX and sulfadiazine-SDZ) and trimethoprim (TMP), are removed through biodegradation pathway (Oberoi et al. 2019). Biodegradation is the breakdown of complex organic compounds such as antibiotics either through biotransformation, resulting in the formation of different metabolic intermediates (i.e., transitory intermediates and/or end products) (Ricken et al. 2013; Reis et al. 2018) or through complete mineralization to H_2_O and CO_2_ by microbial cultures (Bouju et al. 2012; Alvarino et al. 2016; Ricken et al. 2017). Different intermediate compounds may be formed either by hydroxylation, acetylation of the amino group in the case of sulfamethoxazole (Larcher and Yargeau 2011; Zhang et al. 2016; Reis et al. 2018), or breakdown of the parent antibiotic compound (Ricken et al. 2013; Alvarino et al. 2016; Jia et al. 2017; Nguyen et al. 2018). For tetracycline molecules, there is loss of the N-methyl group by demethylation of the dimethyl amino group at the C4 position without breakdown of the parent compound (Leng et al. 2016). Microorganisms are able to degrade pharmaceutical antimicrobials and utilize them as a sole carbon and energy source and/or via co-metabolism (Larcher and Yargeau 2011; Nguyen et al. 2018; Wang and Wang 2018). The biotransformation mechanisms of two classes of antibiotics, namely sulfonamides (SMX) and tetracycline (TET), through biotransformation and mineralization by microorganisms under different redox conditions, intermediates, pathways, catabolic enzymes and genes involved are briefly presented.

### Sulfonamides

**Fig. 2:**
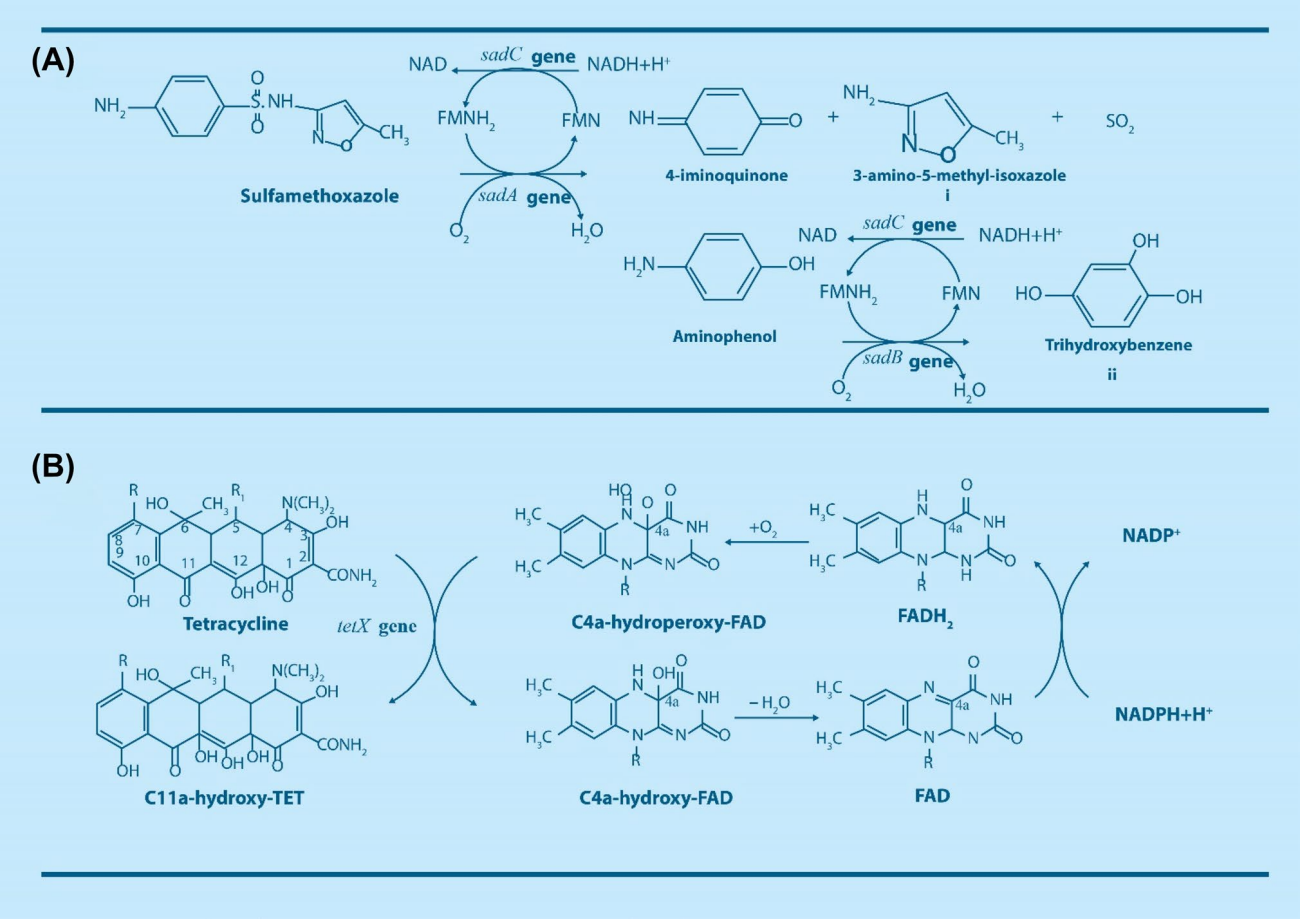
Biodegradation products and pathways of **a** sulfamethoxazole biodegradation encoded by *sad* genes, and **b** tetracycline biodegradation encoded by *tetX* gene

Sulfamethoxazole (SMX), which is among the most frequently detected sulfonamides in the environment, is poorly adsorbed on biological sludge during wastewater treatment. However, biotransformation and mineralization have been observed with both pure and mixed cultures in different redox (aerobic, anoxic, and anaerobic) conditions (Mohatt et al. 2011; Bouju et al. 2012; Kassotaki et al. 2016; Jia et al. 2017; Wang and Wang 2018). Pure bacterial strains such as *Microbacterium* sp. strain BR1(Ricken et al. 2013, 2015), *Achromobacter denitrificans* PR1(Reis et al. 2014), *Pseudomonas psychrophila* HA-4 (Jiang et al. 2014), and *Acinetobacter* sp. (Wang and Wang 2018), have demonstrated the ability to degrade SMX as a sole carbon and energy source under aerobic conditions. In aerobic process involving pure and mixed cultures, sulfamethoxazole is biotransformed to 3-amino-5-methyl-isoxazole (3A5M) (Fig. 2a(i)) (Ricken et al. 2013; Reis et al. 2014; Deng et al. 2016; Mao et al. 2018). The intermediate 3A5M is formed due to the release of 4-iminoquinone and sulfur dioxide from the parent compound (SMX). This ipso-hydroxylation reaction is catalyzed by monooxygenase encoded by the *sad*A gene, allowing separation of the sulfonamide functional group from the parent compound and rendering the intermediates less harmful to the environment (Majewsky et al. 2014; Ricken et al. 2017). In *Microbacterium* sp. strain BR1, a flavin dependent monooxygenase encoded by the *sad*A gene and a flavin reductase encoded by the *sad*C gene are in charge of the initial breakdown of sulfonamide molecules, resulting in the release of 4-aminophenol and its subsequent transformation into 1,2,4-trihydroxybenzene by monooxygenase encoded by the *sad*B gene and flavin reductase encoded by the *sad*C gene prior to mineralization as shown in Fig. 2a(ii) (Ricken et al. 2013, 2017). These reports demonstrate that *Microbacterium* sp. strain BR1 is capable of utilizing sulfonamides for growth and has the capacity to mineralize SMX.

### Tetracyclines

Tetracyclines (tetracycline, oxytetracycline, and chlortracycine) are broad spectrum antibiotics commonly used in livestock production. They are poorly biodegradable due to their complex chemical structures; However, numerous studies have explored chemical processes (i.e., photochemical and electrochemical technologies) for their degradation (Bautitz and Nogueira 2007). It has been shown that tetracyclines may be transformed to C11a-hydroxy-tetracyclines catalyzed by a flavin monooxygenase encoded by *tet*X genes in microbes (Fig. 2b) **(**Markley and Wencewicz 2018**)**. A bacterial strain, *Stenotrophomonas maltophilia* DT1, capable of degrading TET, has been isolated from TET contaminated sites (Leng et al. 2016). Based on the molecular mechanism of TET biotransformation by *S. maltophilia* strain, the nodulation protein efflux pump transported TET outside cells, and hypoxanthine-guanine phosphoribosyl-transferase facilitated the activation of the ribosomal protection proteins. In the end, TET biotransformation was catalyzed by the enzymes superoxide dismutase and peroxiredoxin (Leng et al. 2017).

## Antibiotics promote the evolution and transmission of resistance

Significant genetic variation is associated with mechanisms of genetic exchange occurring frequently among microbial populations and communities spurred by habitats that provide suitable biological interconnection, generate variation, and offer chances for specific selection, leading to the genetic evolution of resistant bacteria (Baquero et al. 2008). Mutation is a key event that can form the basis for the selection of resistance in the mix of bacteria and antimicrobial compounds in the various habitats. Mutations drive antibiotic resistance by occurring spontaneously in the bacterial genome, and the mutants propagate the resistance to the subsequent progeny through vertical evolution and natural selection created by antibiotic pressure (Baquero et al. 2008).

**Fig. 3:**
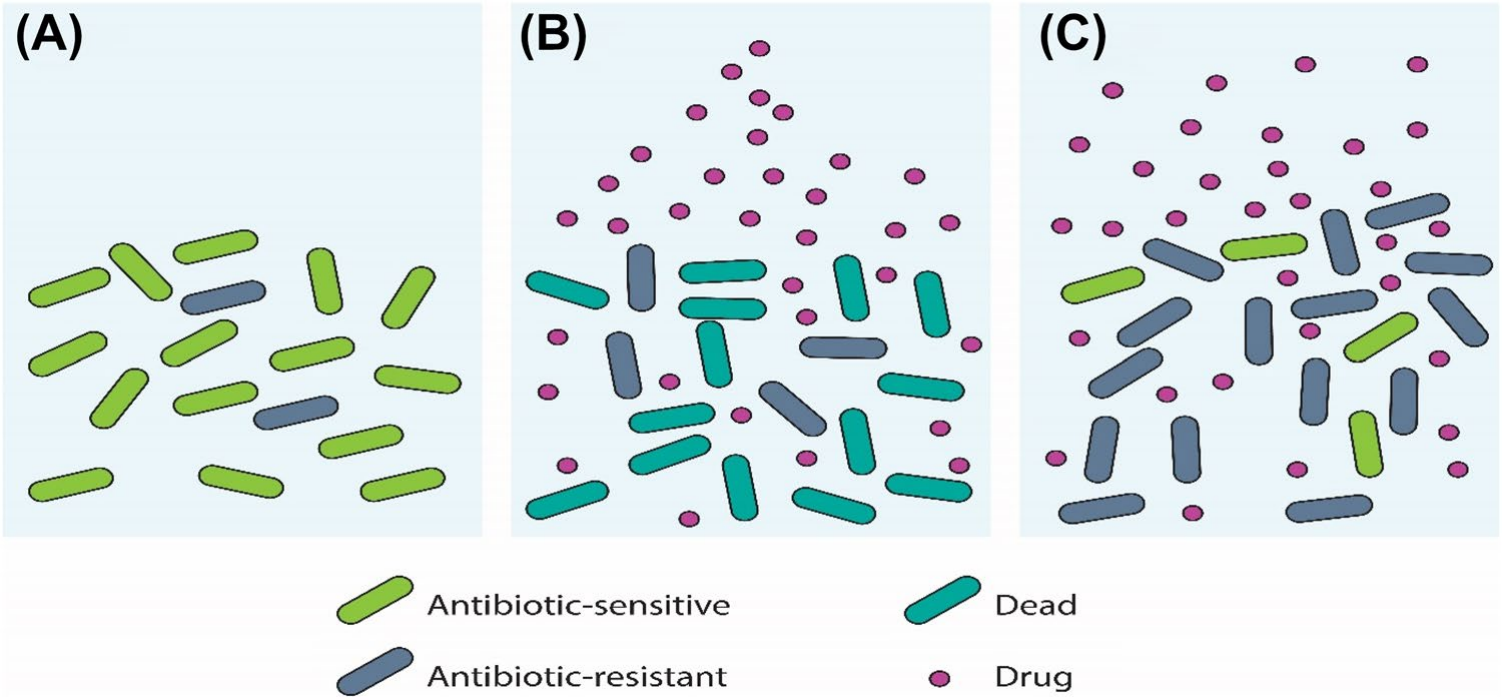
Resistance evolution driven by the presence of antibiotics. **a** Mutant bacteria occur frequently in large population sizes. The frequency of mutants is low in the absence of antibiotics since resistance typically imposes a fitness cost. **b** Resistant bacteria divide faster than sensitive bacteria in an environment created by the presence of antibiotics. **c** Resistant bacteria finally dominate the population, and the antibiotic becomes ineffective

Figure 3 illustrates the role of antibiotics in the selection and proliferation of resistant bacteria driven by mutation.

Since DNA replication is not perfect, cell division may result in random changes to the DNA sequences of descendent cells. The biological effects of the resultant mutations on the cells that carry them can range from insignificant to disastrous. Some mutations, for example, alter the cellular proteins that are frequently targeted in antibiotic treatment. A random mutation that alters a cellular protein required for a specific antibiotic to enter the cells of its target bacterial species blocks the antibiotic entry into the mutant cell and interferes with protein synthesis. Unlike in the absence of antibiotics in which an antibiotic resistance mutation does not provide a selective advantage to a cell, in the presence of antibiotics, the mutant reproduces normally. In the presence of the antibiotics, wild-type drug-sensitive cells would either fail to reproduce or die (Genereux and Bergstrom 2005). Typically, antibiotics designed to kill bacteria end up selecting for bacteria that do not respond to the antibiotics.

**Fig. 4:**
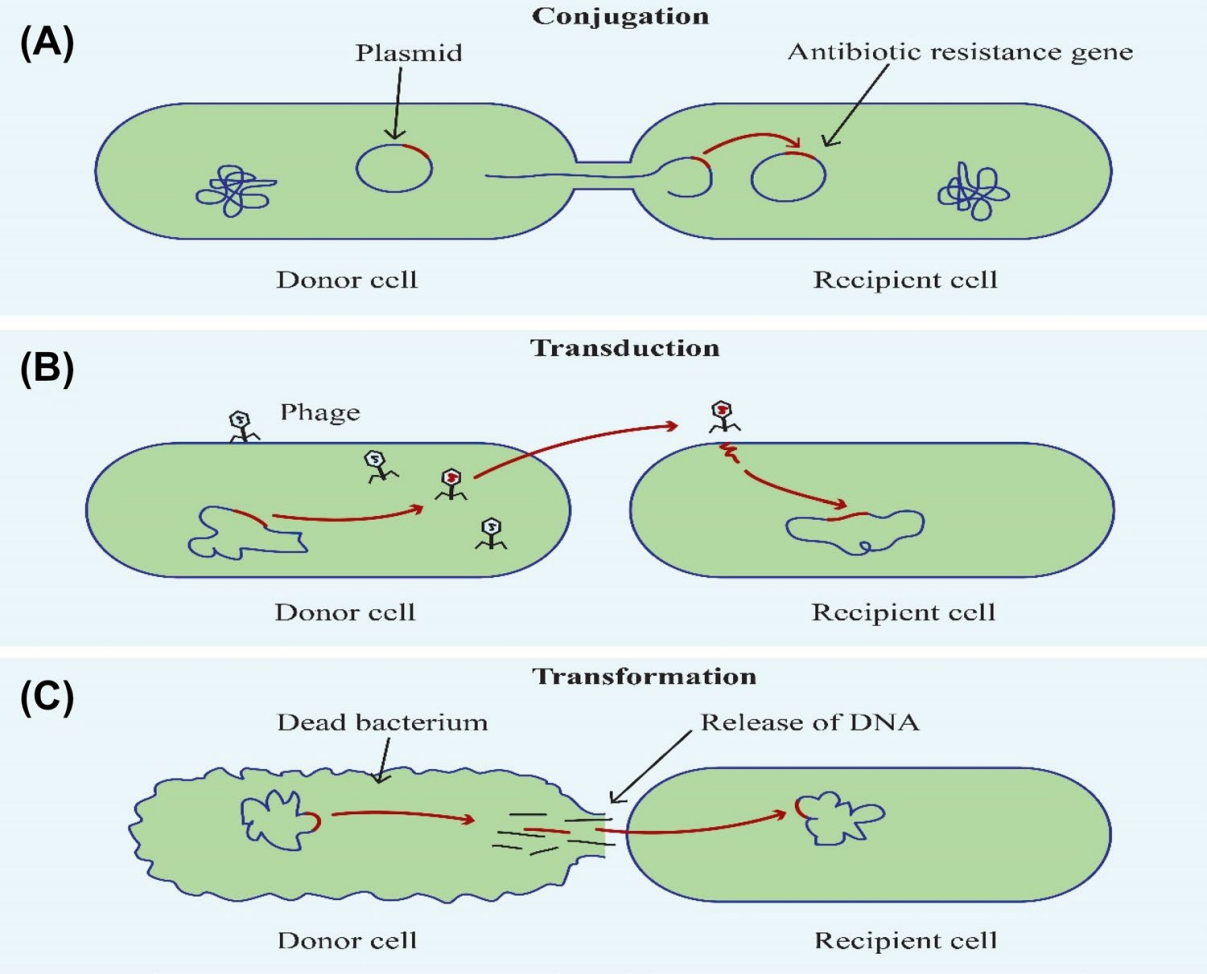
Mechanisms of horizontal gene transfer where bacterial DNA can be transferred from one bacterium to another. **A** Conjugation involves direct contact transfer of mobile plasmids between the donor cell and the recipient cell. **B** Transduction refers to the transfer of DNA from one bacterium to another mediated by bacteriophages. **C** In transformation bacteria pick up free fragments of DNA from the environment and integrate them into their genome

Antibiotic resistance can also be driven by horizontal evolution through gene exchange mechanisms occurring in intra and inter-species (Touchon et al. 2017). Conditions within the environment, especially the WWTPs provide cell proximity, which favors horizontal evolution (or lateral gene transfer). Horizontal gene transfer (HGT) follows either or a combination of the three routes (conjugation, transformation, and transduction) where genetic material is obtained from antibiotic resistant bacteria in each case, and the recipients become resistant. Conjugation involves direct contact transfer of mobile plasmids between the donor cell and the recipient cell. During transformation, bacteria pick up free fragments of DNA from the environment and integrate them into their genome. Transduction refers to the transfer of DNA from one bacterium to another mediated by bacteriophages (Von Wintersdorff et al. 2016) (Fig. 4). Lateral transfer of genetic material occurs frequently among bacterial populations aided by resistance plasmids (R-plasmids), which contain antibiotic resistance genes and have been linked to global antibiotic resistance spread in the vast majority of Gram-negative bacteria (Berglund 2015).

**Fig. 5:**
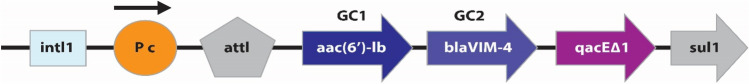
Structure of *In*238 is shown as an example for a typical class 1 integron that consists of two conserved genes at the 3′ end, quarternary ammonium compound resistance gene *qacE∆1* and sulphonamide resistance gene *sul1*. The gene *intI1* encodes a site-specific integrase which is capable of excising and integrating gene cassettes at the site-specific integration site *att1. In*238 contains two gene cassettes designated as GC1 carrying an amino acid modifying enzyme (*aac (6′)-lb*) and GC2 carrying beta-lactamase (*blaVIM-4*). The promoter PC induces the expression of the gene cassettes

Genetic elements (plasmids, transposons, integrons and gene cassettes) that carry genes have high mobility and are easily transferable between strains and, in some cases, taxonomic classes (Von Wintersdorff et al. 2016). Transposons are perfect vehicles for transmitting antibiotic resistance genes within and between microbial populations because of their unique ability to jump from one genetic locus into another where they integrate into a bacterial chromosome and/or plasmids, regardless of their phylogeny (Mazel 2006). Integrons are genetic elements which aid the accumulation of antimicrobial resistance genes. Class I integrons (Fig. 5) consist of an integrase gene, a recombination site and a promoter at the 5′ conserved sequence, and a truncated *qacE* (*qacE∆*) and *sul1* gene at the 3′ conserved sequence. Between the 5′ and 3′ conserved sequences, gene cassettes can be found. The integrase has the ability to capture and integrate antimicrobial resistance genes into the gene cassettes. The promoter of the integron contributes to the expression of genes located in the gene cassettes. The *aacE∆* and *sul1* genes are responsible for resistance to quaternary ammonium compounds and sulfonamides, respectively (Labbate et al. 2009).

ARGs may be subject to HGT in the WWTP and thereby contribute to the spread of ARGs and multi-resistant microorganisms (Du et al. 2015). It has been observed that different genes encoding specific antibiotics are frequently found in the same position on chromosomes or mobile genetic elements, resulting in multiple resistance (Xu et al. 2017). This makes mobile genetic elements such as plasmids, transposons, and integrons crucial in the emergence and spread of ARGs (Zhu et al. 2013). The multi-gene cassettes carried in integrons can encode different ARGs under a mutual promoter and aid in ARG co-selection; thus, selection pressure applied by one antibiotic may select for resistance associated with multiple ARGs found in the integron’s gene cassettes (Di Cesare et al. 2016). Methicillin-resistant *Staphylococcus aureus* (MRSA), for example, acquires a gene cassette that transfers multiple ARGs simultaneously (Sharma et al. 2016).

## Resistance mechanisms and occurrence of ARGs in wastewater

**Fig. 6:**
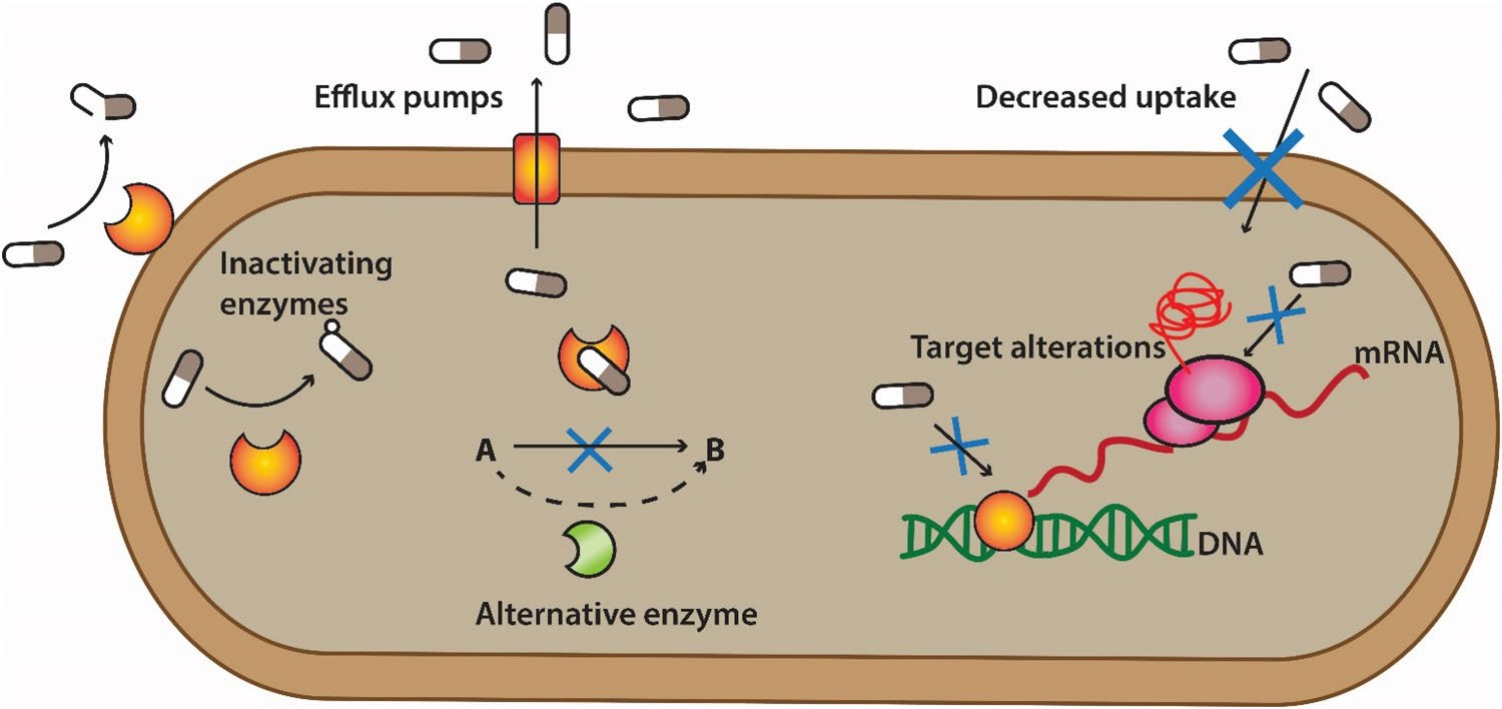
The mechanisms of antibiotic resistance in bacteria. Acquired enzymes inactivate the drugs, active efflux pumps transport specific or multiple antibiotics out of the cell, alternative metabolic pathways substitute those inhibited by the drug, modification of antibiotic target site leads to reduction of drug affinity to the binding sites and decreased drug accumulation due to decreased permeability

Despite the presence of diverse antimicrobial pharmaceuticals in the aquatic environment that could possibly inhibit the growth of bacteria, many studies have demonstrated the presence of both antibiotic- resistant bacteria and antibiotic resistance genes in the same environments. The compounds target different sites of the bacterial cell and exert their action either with a bactericidal or bacteriostatic effect. In order to counteract the effect of antimicrobials and survive environmental stress, bacteria have evolved various defense mechanisms. The most common resistance mechanisms are: (1) alteration or modification of the antibiotic target site leading to reduction of drug affinity to the binding sites like the modified penicillin binding proteins (PBPs), (2) decreased drug accumulation due to decreased permeability or to expression of active efflux pumps which transport specific or multiple antibiotics out of the cell (Munita and Arias 2016), (3) use of acquired or endogenously produced enzymes to inactivate the antibiotics, and (4) acquisition of alternative metabolic pathways to substitute those inhibited by the drug (Kumar et al. 2019) (Fig. 6).

The human gut resistomes are dominated by particular ARG types with high prevalence. Genes conferring resistance toward tetracycline, aminoglycoside, beta-lactam, macrolide-lincosamide-streptogramin (MLS), and vancomycin are more abundant compared to resistance gene types such as bacitracin, chloramphenicol, fosmidomycin, and polymyxin, while six tetracycline resistance genes (*tet32*, *tetM*, *tetO*, *tetQ*, *tetW*, and *tet resistance protein*) are quite common, indicating widespread occurrence in the human gut (Qiu et al. 2020). In a study by Yan et al. investigating the distribution of antibiotic resistance genes in monkey gut microbiota, 9 types of resistance genes were found in human gut microbes, with 11 types of resistance genes occurring in both humans and cynomolgus monkeys. Among them, the bacteria-harboring resistance genes to bacitracin, tetracycline, and macrolide-lincosamide-streptogramin accounted for a high proportion in both humans and cynomolgus monkeys (Yan et al. 2022). Although glycopeptide, aminoglycoside, beta-lactam, sulfonamide, and macrolide-lincosamide-streptogramin resistance genes occur frequently in humans and cynomolgus monkeys’ gut, tetracycline resistance gene, *tet(37)*, has been found to be the most widespread and dominant ARG in metagenomic resistome profiles of humans and cynomolgus monkeys (Yan et al. 2022). In chicken gut, genes coding for resistance to tetracycline, macrolide-lincosamide-streptogramin B (MLS) antibiotics, and aminoglycosides were found to be more prevalent (Juricova et al. 2021). Data from various sources indicated that many of these ARGs, which are associated with the resistance mechanisms (e.g., target protection, target modification, drug modification, reduced permeability or efflux) were found in environmental samples, suggesting that their origin may be traced to humans and animal sources. The data encompasses both examples of genes detected in cultured bacterial isolates or total DNA isolated from aqueous environment. Both culture-based and molecular-based techniques are commonly used to study antibiotic resistance in environmental matrices. Although culture-dependent methods have limitations when handling environmental bacteria (as culturable fraction is only 1% of the total), they are important for understanding isolate phenotypic characteristics and resistance profiles. Total DNA is isolated from the samples (e.g., influent, effluent, or activated sludge) and specific nucleotide sequences coding ARGs are detected using polymerase chain reaction (PCR) and/or quantitative polymerase chain reaction (qPCR) techniques to identify specific DNA targets in unculturable microorganisms or those that multiply slowly but significantly contribute to resistance (Pazda et al. 2019). Therefore the ARGs presented in this review have been identified in aqueous environments by one or a combination of the following techniques, culture-dependent, culture-independent, high-throughput sequencing, DNA microarray or Shotgun metagenomic sequencing.

### Resistance to β-lactams

β-Lactams include penicillin derivatives (penams), cephalosporins (cephems), carbapenems, and monobactams that interfere with the synthesis of the bacterial cell wall. β-Lactams inhibit the penicillin binding proteins (PBPs), which catalyze the transpeptidation process during peptidoglycan synthesis and thus prevent the cross-linking that forms a cell wall structure that is closely knit. The common mechanisms of β-lactam resistance are alteration of target sites (mutations in PBPs) and direct deactivation by β-lactamases (Tang et al. 2014). Enzymatic inactivation is the key resistance mechanism involving the expression of β-lactamase enzymes encoded by *bla* genes either on a plasmid or chromosomal DNA, β-lactamases cleave the β-lactam ring and inactivate or degrade the antibiotic (Deshpande et al. 2004). *bla* genes commonly transferred via mobile genetic elements often coexist with other resistance genes, which are co-transmitted in the environment, increasing the chances of multidrug resistance (Tennstedt et al. 2003; Schluter et al. 2007). Many different types of β-lactamases confer resistance to the most clinically important β-lactams where a single amino acid change may affect the substrate specificity of the enzyme. β-lactamases can be categorized (1) into classes A–D based on Ambler molecular classification or (2) according to the Bush-Jacoby (functional) grouping (Ambler 1980; Bush and Jacoby 2010).

### Extended spectrum β-lactamases

Extended spectrum β-lactamases (ESBLs) form a group of enzymes that confer significant resistance to penicillins, aminopenicillins, oxyimino-cephalosporins (such as ceftazidime, cefotaxime, ceftriaxone, cefepime), and monobactam (aztreonam) and their activity is inhibited by β-lactamase inhibitors such as clavulanic acid (Coque et al. 2008). ESBL types are diverse, but three types are the most common. The ESBL type TEM β-lactamases are mostly found in Gram-negative bacteria, especially in *Escherichia coli* and *Klebsiella pneumonia*e. These are enzyme derivatives of non-ESBL TEM-1 and TEM-2, in which TEM-1 was first reported in 1965 from an *E. coli* isolate from a patient in Athens, Greece, and since then, about 140 TEM types have been described (Bradford 2001). TEM variants have been recovered in influent, activated sludge, and effluent of a WWTP (Korzeniewska and Harnisz 2013; Biswal et al. 2014; Neudorf et al. 2017). SHV-1 type is a non-ESBL β-lactamase that shares structural similarity and sequence similarity of 68% with TEM-1 and mostly occurs in *K. pneumonia*e. Although more than 60 SHV variants have been identified, SHV-5 and SHV-12 are the most common variants (Jacoby and Munoz-Price 2005). The *bla*_SHV_ gene has been detected in activated sludge and the effluent of WWTPs (Szczepanowski et al. 2009; Marti et al. 2013). The genes coding for CTX-M β-lactamase enzymes are frequently plasmid acquired and show 40% sequence similarity with the genes coding for TEM or SHV β-lactamases. The CTX-M group includes more than 80 variants mostly found in the strains of Enterobacterales and confers significantly higher resistance against cefotaxime compared with other oxyimino-cephalosporin substrates such as ceftazidime, ceftriaxone, or cefepime (Partridge 2015). *bla*_*CTX−M*_ variants have similarly been identified in influent, activated sludge, and the effluent of WWTPs (Szczepanowski et al. 2009; Korzeniewska and Harnisz 2013; Biswal et al. 2014). The OXA-type enzymes, which belong to the molecular class D, differ entirely from TEM and SHV enzymes. Certain OXA variants belong to the family of ESBLs according to their substrate profile. The *bla*_*OXA*_ genes show 20% sequence similarity with the other genes coding for ESBLs, and such genes have been recovered in the activated sludge and the effluent of WWTPs (Szczepanowski et al. 2009; Yang et al. 2013).

### AmpC cephalosporinases

Besides ESBL enzymes, which are the most common forms of acquired resistance to broad-spectrum cephalosporins, class C β-lactamases, referred to as AmpC-type enzymes, can confer high-level resistance to those antimicrobial agents as well. The most common plasmid-encoded AmpC enzymes are CMY-, DHA- and ACC-type β-lactamases, with CMY-type enzymes having a higher prevalence worldwide (Arlet and Jacoby 2002). The production of endogenous AmpC β-lactamase (chromosomal cephalosporinase) can be induced by several β-lactams including benzylpenicillin and narrow-spectrum cephalosporins (Hooper and Gordon 2001). The enzyme is usually produced in low quantities (low-level expression) and determines resistance to aminopenicillins (ampicillin and amoxicillin) and most of the early cephalosporins. *bla*_*CMY*_ was the most common AmpC cephalosporinase gene detected in the effluent and the activated sludge of WWTPs (Szczepanowski et al. 2009).

### Carbapenemases

Carbapenems, which include imipenem, meropenem, ertapenem, and doripenem, are the most effective β-lactams against Gram-negative bacilli due to their high permeability of bacterial outer membranes, affinity for penicillin-binding proteins, and stability against extended-spectrum lactamases (ESBLs) (Zavascki et al. 2010). The majority of carbapenemases are Class B metallo-β-lactamases (MBL), which contain zinc ions rather than serine in their active site, unlike the serine in classes A, C and D β-lactamases. MBLs confer resistance to carbapenems, usually in addition to other β-lactams except aztreonam, and to clinical β-lactamase inhibitors (Cornaglia et al. 2011). The VIM (Verona integron encoded metallo-β-lactamase), IMP (imipenem resistant pseudomonas) and NDM (New Delhi Metallo-β-lactamase) enzymes and their variants are the most commonly identified as coded on a variety of plasmids and harbored by several strain types and species (Johnson and Woodford 2013). Klebsiella Pneumoniae carbapenemases (KPC) and OXA-48-like carbapenemases belong to class A and class D β-lactamases, respectively (Poirel et al. 2012). *bla*_*IMP*_, *bla*_*VIM*_, *bla*_*KPC*_ and *bla*_*NDM*_, which are common in members of the Enterobacterales, have been detected in enteric bacteria isolated from hospital effluents, activated sludge, and effluent in WWTPs (Alexander et al. 2015; Cahill et al. 2019).

### Resistance to aminoglycosides

Aminoglycosides bind to the aminoacyl-tRNA recognition site (A-site), the decoding centre on the 16S rRNA of the ribosome, thus inhibiting protein synthesis. The most clinically relevant members commonly used against infections caused by Gram-negative bacteria are gentamicin (GEN), amikacin (AMK) and tobramycin (TOB) (Bartlett 2005; Partridge 2015). Resistance development associated with their use is due to acquired inactivation enzymes and 16S rRNA methylases (Poirel et al. 2018).

### Aminoglycoside modifying enzymes (AME)

These enzymes, mostly linked to genes encoded on mobile elements mainly on integrons, confer resistance to aminoglycosides by acetylating, adenylylating or phosphorylating the aminoglycosides (Ramirez and Tolmasky 2010). The aminoglycoside acetyltransferases act by catalyzing the addition of an acetyl group (CH3CO) from acetyl coenzyme A to an amine group (–NH2) at positions 1, 2, 3, or 6 of the aminoglycoside structure, which determines the subset of the enzyme (Dolejska et al. 2013). In Gram-negative bacteria, the most common aminoglycosides nucleotidyltransferases are ANT(2″) and ANT(3″) encoded by the genes *aadB* and *aadA*, respectively, both of can be part of gene cassettes carried in class 1 integrons while Streptomycin resistance is mediated by APH(6)-Ia and APH(6)-Id aminoglycoside phosphotransferases encoded by the *strA* and *strB* genes respectively (Ramirez and Tolmasky 2010). More than 50 genes encoding AME have been described, but many variants of the gene cassette-borne acetyltransferases (AAC) appear to dominate in clinically important Gram-negative bacteria (Partridge et al. 2009). Variants of aminoglycoside modifying enzymes such as AAC1, AAC2, AAC4 and APH(6) have been detected in bacteria isolated from hospital wastewater and in both the influent and effluent of WWTPs (Khan et al. 2019).

### 16S rRNA methyltransferases (RMTases)

These are mainly plasmid-borne 16S rRNA methyltransferases (RMTases) which promote target protection by methylating the 16S rRNA of the 30S ribosomal subunit at the A site, which interferes with aminoglycoside binding and results in high-level resistance to aminoglycosides (Wachino and Arakawa 2012). Various 16S rRNA methylases originating from natural aminoglycoside producers as self-protection against these antimicrobials such as ArmA, RmtA/B/C/D/E/F/G/H, and NmpA, have been identified in Gram-negative bacteria including *Acinetobacter baumannii*, Enterobacterales and *Pseudomonas aeruginosa* isolates (Yu et al. 2007; Batah et al. 2015). ArmA, RmtB RmtC and RmtF are the most commonly identified enzymes in Enterobacterales, while ArmA RmtA, RmtB and RmtD are the 16S rRNA methylases promoting aminoglycoside resistance described in *P. aeruginosa* (Jin et al. 2009; Lincopan et al. 2010; Zhou et al. 2010). Some of these 16S rRNA methylase encoding genes, particularly *armA* and *rmtB* genes, have been found in municipal wastewater, hospital wastewater and in both influent and effluent of WWTP (Zurfluh et al. 2017).

### Resistance to quinolones and fluoroquinolones

Quinolones and fluoroquinolones act on DNA gyrase and topoisomerase IV enzymes, which have essential roles during DNA replication. While DNA gyrase introduces negative supercoils, topoisomerase IV removes knots in DNA. The enzymes consist of a tetramer with DNA gyrase having two GyrA plus two GyrB subunits and topoisomerase IV consisting of two ParC plus two ParE subunits. The enzymes introduce double-stranded breaks in the DNA, then re-ligate. Quinolones bind to the cleaved-ligated active site, thereby intercalating into the DNA and blocking the ligation process, resulting in DNA fragmentation, which impairs the function of the two enzymes (Aldred et al. 2014).

### Chromosomal (Fluoro) quinolones resistance by target site mutations

Mutations usually occur in the gyrase gene, the preferred target of quinolones in Gram-negative bacteria, while additional mutations in the topoisomerase IV gene in some highly resistant isolates have been described (Jacoby 2005) (2). These mutations are found in the “quinolone resistance determining region” (QRDR) of *gyrA* and/or *parC* (Drlica et al. 2009). Mutations in *gyrB* and *parE* are not common, but multiple mutations in *gyrA* and/ or *parC* have been described in highly resistant isolates of fluoroquinolone-resistant *P. aeruginosa* (Muramatsu et al. 2005; Rejiba et al. 2008). Mutations in the DNA gyrase and topoisomerase IV enzymes conferring high-level resistance to fluoroquinolones, especially ciprofloxacin, have also been observed in Enterobacterales (Drlica et al. 2009; Tam et al. 2010). Genes encoding these enzymes, which occur naturally in the bacterial chromosome, particularly *gyrA* and *parC*, are found in influent, effluent and activated sludge (Xu et al. 2015).

### Plasmid-mediated (Fluoro) quinolones resistance

Several *qnr* genes, including A, B, C, D, S, and VC families that occur on plasmids, encode proteins that prevent quinolones from entering cleavage complexes by binding to DNA, which decreases the action of DNA gyrase and topoisomerase IV, with the resultant effect of low level fluoroquinolone resistance (Strahilevitz et al. 2009; Aldred et al. 2014). Among the most common of these proteins are *qnrB* genes, which are derived from chromosomes of different *Citrobacter* species (Jacoby et al. 2011), *qnrA* genes derived from *Shewanella* algae, *qnrD* genes mostly linked to small plasmids in *Proteus mirabilis*, and *qnrS* genes common in *Vibrio splendidus* (Poirel et al. 2005), Both *qnrA* and *qnrB* occur frequently on class 1 integrons where they are co-carried in association with other resistance determinants (Robicsek et al. 2006). The occurrence of *qnr* genes in an aqueous environment is common. *qnrS* was detected in the activated sludge (Bönemann et al. 2006). Forcella et al. observed *qnrB* genes in wastewater effluent from a WWTP (Forcella et al. 2010), while *qnrB* and *qnrS* were identified in soil that had been irrigated with wastewater (Dalkmann et al. 2012). The genes *qnrA*, *qnrB*, and *qnrS* were detected in a wetland along an urban coast bordering the United States and Mexico (Cummings et al. 2011). *qnrC* and *qnrD* have been found in activated sludge and effluent from a WWTP (Xu et al. 2015), whereas *qnrA* and *qnrB* have been found in a WWTP’s effluent (Marti et al. 2013).

### Resistance to sulfonamides and trimethoprim

The combination of sulfamethoxazole and trimethoprim acts by interfering with the two successive steps in folate biosynthesis. Sulfonamide resistance is achieved by genes encoding drug-resistant dihydropteroate synthases, *sul1*, *sul2*, or *sul3*. The *sul1* gene forms part of the 3′-conserved segment of class 1 integrons and is often transmitted together with other ARGs occurring on gene cassettes in the variable region (Recchia and Hall 1995). The *sul2* gene frequently occurs on plasmids that harbor other ARGs. The *sul3* gene is often associated with unusual class 1 integrons and has been linked to the macrolide resistance gene *mef* (B) (Sunde et al. 2008; Liu et al. 2009; Siqueira et al. 2016). *Sul1* and *sul2* have been found in Australian and German surface waters (Stoll et al. 2012), and in freshwater and marine waters in the Philippines (Suzuki et al. 2013). *Sul1* has also been found in wastewater (Gao et al. 2012; Berglund et al. 2015). *Sul1, sul2*, and *sul3* were all found in effluent and activated sludge (Szczepanowski et al. 2009). Trimethoprim resistance genes are categorized as *dfrA* and *dfrB*, with at least 19 different *dfrA* variants and less than 8 different *dfrB* gene cassettes, which encode trimethoprim-resistant dihydrofolate reductases. These genes were described in Enterobacterales and other Gram-negative bacteria (Partridge et al. 2009). Most *dfrA* and *dfrB* genes occur on gene cassettes integrated into class 1 or class 2 integrons. A few other *dfrA* genes are associated with ISCR1 or ISCR2 elements. The occurrence of a *dfrA* gene linked with ISCR1 and *sul1* in the 3′-CS in a class 1 integron can confer resistance to trimethoprim/sulfamethoxazole (cotrimoxazole). *Sul3* is linked to a type of class 1 integron only known to be associated with a gene cassette that includes *dfrA12* (Partridge et al. 2009). The occurrence of *dfr* genes in the environmental matrices is common. *dfrA1, dfrA7, dfrA12*, and *dfrA17* were found as part of integrons in a polluted lagoon in Portugal (Henriques et al. 2006), and *dfrA1* and *dfrA12* were found in a WWTP with a connection to a slaughterhouse (Moura et al. 2007). *drfA1* has also been detected in surface waters in Germany and Australia (Stoll et al. 2012). *dfrA* has been reported in both the influent and effluent of a WWTP (Bengtsson-Palme et al. 2016), while *dfrA* and *dfrB* have been detected in the activated sludge (Szczepanowski et al. 2009).

### Resistance to tetracyclines

Tetracyclines bind to the 30S ribosomal subunit and interfere with the association of aminoacyl-tRNA, inhibiting bacterial protein biosynthesis (Yang et al. 2005). They are widely used in veterinary medicine, accounting for 37% of the total sales of veterinary antimicrobial agents in the European Economic Area (Grave et al. 2014). Due to their widespread use, they have imposed selective pressure on bacteria, leading to the development of resistance. Tetracyclines resistance is through ribosomal protection mediated by large proteins coded by genes such as *ortA*, *tetB(P)*,* tetM*, *tetO*, *tetQ*, *tetS*, *tetT*, *tetW*, *tetX* and decrease in intracellular drug concentration achieved through active efflux from the cell due to proteins coded by genes such as. *tetA*, *tetA(C)*,* tetA(P)*,* tetB*, *tetC*, *tetD*,* tetE*, *tetG*, *tetH*, *tetJ*, *tetK*,* tetL*,* tetV*,* tetY*,* tetZ* (Pazda et al. 2019). A rarely observed mechanism is enzymatic inactivation of the antibiotic coded by the gene *tetX* (Aminov et al. 2001). Tetracycline resistance genes are carried on bacterial chromosome (e.g. *tetA(P)*,* tetA(2)*,* tetJ*,* tetQ*,* tetV*), plasmids (e.g. *tetA*,* tetA(C)*,* tetC*,* tetD*,* tetE*,* tetK*,* tetY*), transposons and ICEs (e.g. *tetB*,* tetH*,* tetM*) (Pazda et al. 2019). Some of the genes may be found on both the chromosome and the integron (e.g. *tetG*) or on the chromosome and plasmid (e.g. *tetL*) (Tuckman et al. 2007). Many of the mobile genetic elements carrying *tet* genes are conjugative and co-transmit genes encoding resistance to other antibiotic compounds. The diversity of mobile elements that mobilize *tet* genes includes plasmids, transposons, integrons, and ICEs, and contributes significantly to the transmission of tetracycline resistance in different bacterial genera (Roberts 2005). Due to their widespread presence, *tet* genes including *tetA*,* tetB*,* tetC*,* tetD*,* tetE*,* tetG*,* tetM*,* tetO*,* tetS*, and *tetQ* have been identified in wastewater from two WWTPs in Wisconsin, USA (Auerbach et al. 2007). *tetA*,* tetC*,* tetG*,* tetM*,* tetS*, and *tetX* have been detected in activated sludge from different wastewater treatment plants (Zhang and Zhang 2011) (3). *TetA*,* tetB*, and *tetC* have been found in a WWTP’s influent, activated sludge, and effluent (Jacoby 2009; Zhang et al. 2009b; Xu et al. 2015). *tetD* and *tetE* were found in WWTP influent, activated sludge, and effluent (Szczepanowski et al. 2009; Jiao et al. 2018).

### Resistance to macrolides

Macrolides bind to the 50S subunit of the bacterial ribosome and inhibit protein synthesis during the early stages (McArdell et al. 2003; Göbel et al. 2005). Bacterial resistance to macrolide antibiotics occurs through several mechanisms which include: (1) the synthesis of methylase enzyme coded by *erm* genes which methylate 23S rRNA, the target site of the antibiotic’s action; (2) antibiotic inactivation by modifying enzymes—macrolide phosphotransferases (MPH) coded by *mph* (A) and *mph* (B) genes on plasmids; and (3) active efflux of the drug from the cell coded by genes such as *mefA* and *msrA* (Leclercq 2002). The most widespread of the macrolide resistance genes is *ermB*, which is linked with a variety of different mobile genetic elements such as ICEs located on chromosomes, plasmids and non-conjugative transposons (Roberts 2008). The *ermB* gene occurs along with other antibiotic resistance determinants on a conjugative platform (Roberts and Mullany 2009). *erm* genes such as *ermA*,* ermB*,* ermC*,* ermF*,* ermT*, and *ermX* are prevalent in a variety of different environments and have been found in wastewater in Portugal and surface waters in Germany and Australia (Araújo et al. 2010; Stoll et al. 2012). In a WWTP, *ermB* and *ermF* genes were found in influent, effluent, and activated sludge (Yang et al. 2014), while *mph*(A*)* and *mph*(B) were found in effluent and activated sludge (Szczepanowski et al. 2009). Table 3 shows different antibiotics and some corresponding ARGs detected in the environment.

**Table 3:** Different antibiotics and the respective ARGs found in the environment

Antibiotics classes	Type of ARGs	Detected environment
Tetracyclines	*tetA*,* tetA(C)*,* tetB*,* tetC*,* tetE*,* tetF*,* tetH*,* tetK*,* tetL*,* tetM*,* tetN*,* tetO*,* tetQ*,* tetS*	Sewage, sludge, surface water, fish ponds, natural water bodies (Cheng et al. 2020), activated sludge (Bengtsson-Palme et al. 2016; Zhang et al. 2016; Jiao et al. 2018)
Macrolides	*mphA*,* mphB*,* ereA2*,* ermA*,* ermB*,* ermF*,* ermO*,* mefA*	Natural water bodies (Cheng et al. 2020), influent, activated sludge, effluent (Bengtsson-Palme et al. 2016; Jiao et al. 2018; Sabri et al. 2020)
Sulfonamides and trimethoprim	*sulI*,* sulII*,* sulIII*,* dfrA*,* dfrB*	Natural water bodies (Oberoi et al. 2019), influent, activated sludge, effluent (Bengtsson-Palme et al. 2016; Neudorf et al. 2017; Sabri et al. 2020)
Beta-lactams	*tem*,* shv*,* ctx*,* ampR*,* cit*,* ges*,* nps*, *sme*, *veb*	Various environment (Oberoi et al. 2019), influent, activated sludge, effluent (Zhang and Li 2011, Yang et al. 2013, Biswal et al. 2014, Amador et al. 2015)
Quinolones	*gyr(A, B)*,* qnrA*,* par(C, E)*,* qnrB*,* qnrC*,* qnrS*	Natural water bodies (Oberoi et al. 2019), influent, effluent, activated sludge (Marti et al. 2013; Xu et al. 2015)
Chloramphenicols	*catI*,* catII*	Natural water bodies (Oberoi et al. 2019)
Glycopepetides	*vanA*,* vanB*,* vanC1*,* vanC2*, *vanC3*,* vanD*	Natural water bodies (Oberoi et al. 2019)
Multidrug efflux pump genes	*amrB*,* mdtG*,* mdtH*,* mexD*,* qacE∆1*	Influent, activated sludge, effluent (Yang et al. 2013, 2014; Jiao et al. 2018)

## Environmental antimicrobial contamination and correlation to antibiotic resistance

Antibiotic concentrations in wastewater have been reported ranging from ng L^−1^ to mg L^−1^, depending on the source: hospitals, urban wastewater, and/or WWTPs (Segura et al. 2009; Hughes et al. 2013). Despite their occurence, studies have not yet established the effect of antibiotic levels on bacteria in the environment, although antibiotic concentrations lower than the minimal inhibitory concentration (MIC) (15,000 ng L^−1^ for tetracycline, and from 2500 ng L^−1^ to as low as 100 ng L^−1^ for ciprofloxacin) have been shown in vitro to select for antibiotic resistant bacteria based on mutations carried by specific strains (Gullberg et al. 2011). In comparison with laboratory experiments, physicochemical parameters and other dynamics in a complex environmental community may influence the concentrations required for selection. However, the negative effects of antibiotic contamination are likely to manifest at different concentrations found in wastewater. Curiously, studies have shown that conjugation and recombination events in bacteria can be induced by certain antibiotics even at sub-inhibitory concentrations (Barr et al. 1986; Úbeda et al. 2005), confirming the role of HGT in the transfer of ARGs in antibiotic-contaminated environments. According to some studies, environments exposed to extremely high levels of antibiotic contamination, especially effluent from pharmaceutical manufacturing facilities, show increased antibiotic resistance indicators (Berglund 2015). For example, effluent from an oxytetracycline-manufacturing plant in China was found to contain high levels of oxytetracycline (Li et al. 2008). In this study, bacterial strains isolated downstream of the river receiving the oxytetracycline waste were more frequently multidrug resistant and had significantly higher MICs against various antibiotic classes compared to those isolated upstream of the river. In addition, many different *tet* genes were found in the isolates obtained downstream (Li et al. 2010). In a similar study, the concentration of ciprofloxacin was more than 1000 times the inhibitory concentration for some bacterial strains in effluent from a WWTP processing wastewater from an antibiotic manufacturing plant in India (Larsson et al. 2007), and the water downstream of the plant contained more abundant ARGs compared to upstream (Kristiansson et al. 2011). Similarly, sulfamethoxazole and trimethoprim concentrations in a river downstream of a pharmaceutical manufacturing facility in Pakistan were found to be 49,000 ng L^−1^ and 28,000 ng L^−1^, respectively, while the corresponding ARGs *sul1* and *dfrA1* concentrations were 0.80 and 0.43 genes/16S rRNA genes, respectively (Guerin et al. 2009). It was established that ARG abundance was correlated with antibiotic contamination. For instance, in a Swedish river, higher ARG concentrations were determined downstream of a WWTP discharging treated effluent from the adjacent city (Berglund et al. 2015), whereas in a river in Colorado, USA, ARG concentrations were found to be higher at sites impacted by anthropogenic activity than at pristine sites upstream (Pei et al. 2006). It remains unclear whether the reported increase in ARG concentrations in these studies was due to anthropogenic activities or proliferation. Nonetheless, the abundance of these ARGs may be an indicator of their involvement in the transformation of bacteria into resistant strains. These findings necessitate advancement of experimental research to decipher the complex interactions involved in antibiotic resistance proliferation driven by antibiotics and dissemination in environmental microbial communities.

## Approaches to combat antimicrobial resistance to complement antibiotics and ARGs removal in WWTPS

Various reviews have adopted an approach to summarize the fight against the spread of antimicrobial resistance (AR) from the point of view of advancing the technologies applied in the removal of antibiotics and ARGs during wastewater treatment (including chlorination, UV disinfection, ozonization, solar photocatalysis, advanced oxidation process, membrane bioreactor, bioelectrochemical system, solar Fenton oxidation) (Barancheshme and Munir 2018; Pazda et al. 2019; Zhu et al. 2021). However, in addition to upgrading these technologies to reduce the resistance determinants, this review explores a divergent approach by presenting the alternative antibiotic therapeutic approaches to minimize AR by reducing or substituting antibiotic use by humans, and in addition, the removal antibiotics from contaminated sites through the bioremediation approach. Whereas stewardship programs are continuously promoted to enhance judicial use of antibiotics in the hospital and community sectors, create awareness on hygiene and effective management of medical wastes, and to limit the use of antimicrobials in animal husbandry among others, the development of novel therapeutic approaches to reduce antimicrobial resistance is highly desirable and continues to receive increased attention. Notable approaches which have shown prospects include the use of phage therapy, vaccine strategy, nanoparticles, and antimicrobial peptides (Chatterjee et al. 2016), as well as the use of natural compounds.

### Phage therapy

Bacteriophages (phages) are viruses that infect and kill bacteria through lysis (Clokie et al. 2011). Phage therapy has several advantages, including replication at the infection site, high specificity to target bacteria without affecting commensal flora, fewer side effects compared to other treatments, bactericidal activity against antibiotic-resistant bacteria, and ease of administration (Chatain-LY 2014). Phages can increase treatment efficacy by being genetically engineered to deliver antimicrobial agents to bacteria (Pires et al. 2016). For instance, a variety of genetically engineered *E. coli* phages were created to degrade biofilms, or target specific DNA sequences involved in antibiotic resistance by delivering RNA-guided nucleases (Citorik et al. 2014). Although phage-infected bacteria can develop resistance to phages over time, the rate of developing resistance to phages is much lower than the rate of developing resistance to antimicrobials (Morris Jr et al. 2001). Instead of using a single type of phage, a mixture of phages may be able to slow the evolution of bacterial resistance to phages (Örmälä and Jalasvuori 2013). Moreover, phages continue to be one of the most abundant genetic resources, and they evolve quickly in response to bacterial resistance (Stern and Sorek 2011), which makes them promising in the control of bacterial infections.

### Vaccine strategy

The vaccine strategy aims to prevent infection before it becomes established, thereby reducing bacterial infections. Notable examples are the potential candidate vaccines that have shown prospects for the control of bacteria such as *P. aeruginosa*, which include, LPS O-antigen, polysaccharide protein conjugates, outer membrane proteins OprF and OprI, the type III secretion system component PcrV, flagella, pili, DNA, and whole killed cells (Döring and Pier 2008). Although vaccines may be effective methods of preventing antimicrobial resistance, impaired host defense mechanisms frequently reduce vaccination efficacy (Baker et al. 2020). Steps towards the achievement of a vaccine strategy against pathogens are being pursued, such as the development of novel *P. aeruginosa* vaccines, which is currently underway. The POH vaccination was found to be effective in protecting mice against clinical *P. aeruginosa* strains (Yang et al. 2017). The development of multivalent vaccines appears promising and may provide a future method of protection against bacterial infections.

### Nanoparticles

Nanoparticles are tiny materials with a surface area to mass ratio of more than 100 nm that have been used in a variety of chemical, biological, and biomedical applications (Jeevanandam et al. 2018). Antimicrobial nanoparticles are currently receiving a lot of attention for the treatment of a variety of diseases, including bacterial infectious diseases, due to several advantages, including high penetrability into bacterial membranes, the ability to disrupt biofilm formation, possessing multiple antimicrobial mechanisms, and are good antibiotic carriers (Wang et al. 2017). Silver nanoparticles, for example, produce silver ions that inhibit DNA synthesis and are effective antimicrobial agents (Wang et al. 2017). Moreover, silver nanoparticles have demonstrated low cytotoxicity to mammalian cells, though further in vivo testing is required (Salomoni et al. 2017). It has been demonstrated that attaching antibiotics to nanoparticle surfaces significantly improves the efficacy of both antibiotics and nanoparticles (Brown et al. 2012). Nonetheless, nanoparticles present certain drawbacks because of their high surface area to mass ratio, which makes them highly reactive and may cause reactions, thus potentially toxic to the human body (Elsaesser and Howard 2012). In addition, they are easily transported to distant organs and can cause systemic toxicity (Yildirimer et al. 2011). Although they may offer an effective alternative to the use of antibiotics, nanoparticles are still restricted to preclinical stage experiments due to their potential side effects and thus, have not yet been introduced into clinical practice.

### Antimicrobial peptides

Antimicrobial peptides, also referred to as host defense peptides, are produced by a variety of organisms and have antimicrobial activity against a wide range of microorganisms (Toke 2005). It is widely assumed that these peptides target the cytoplasmic membrane, resulting in cell death (Park et al. 2011). They have been shown to have anti-biofilm properties in addition to antimicrobial activity (Chung and Khanum 2017). Antimicrobial peptides have been proposed as an alternative to conventional antibiotics to combat bacterial infections due to their broad-spectrum activity, low levels of induced resistance, and low toxicity to the host (Hancock et al. 2016). Antimicrobial peptides promote antibiotic uptake, disrupt biofilm formation, or inhibit bacterial quorum sensing when used with conventional antibiotics to produce synergy against bacteria (Grassi et al. 2017). For example, GL13K combined with tobramycin increased the clearance of *P. aeruginosa* biofilm (Hirt and Gorr 2013). Antimicrobial peptides have the potential to reduce the spread of antimicrobial resistance. However, they are likely to be limited by rapid degradation in the human body due to proteolysis, hemolytic activity in host cells, and reduced activity based on salt and pH sensitivity (Aoki and Ueda 2013).

### Natural compounds

Use of natural compounds has demonstrated potential prospects to control bacterial infections. A variety of natural compounds have been tested against pathogens and shown commendable levels of efficiency. Notable examples include, the combined effect of methicillin and the bacteriocin leaderless two peptides enterocin DD14 (EntDD14), which has recently shown the capability to reduce biofilm formation of *Staphylococcus aureus* S1 by about 30% (Belguesmia et al. 2021). In this study, EntDD14 downregulated the expression of the main genes, *nuc* and *pvl*, involved in biofilm formation, which code for nuclease and Panton-Valentine leucocidin, respectively, known to be responsible for MRSA-S1 virulence. Other genes, such as *cflA*, *cflB*, and *icaB*, which code for bacterial ligand clumping factors A, B, and intercellular adhesion factor, respectively, were also shown to be downregulated, implying that bacteriocins and antibiotics can be used in tandem to treat bacterial infections. The Pantoea Natural Product 2 (PNP-2) and Pantocin A, produced by the clinical strains of *Pantoea agglomerans* Tx10, a cystic fibrosis isolate, have also shown the ability to inhibit the growth of a wide range of multi-drug resistant Gram-negative and Gram positive bacteria, including *Enterobacter*, *E. coli*, *Klebsiella*, *Kosakonia*, *Pseudocitrobacter*, *Salmonella*, *Staphylococcus*, and *Streptococcus* (Robinson et al. 2020). Their broad spectrum of activity suggests potential for the development of a therapeutic strategy to control antimicrobial resistance. In a recent study, some natural compounds belonging to pyrrolidine, anthracyclines, and indole derivatives identified from the actinobacteria, *Streptomyces*, were shown to have inhibitory activity on HIV-1 reverse transcriptase, indicating that actinobacteria are promising sources of biological active metabolites, which could provide important bioactive compounds as potential antibiotic drugs (Hei et al. 2021).

### Bioremediation of antibiotics from contaminated sites

Antimicrobials are discharged into the environment through application in clinical treatment and via animal manure, often contaminating the environment where they are persistent (Bunce and Hellyer 2018; Ezzariai et al. 2018). Bioremediation is the in-situ or ex-situ application of living organisms to detoxify and/or extract chemical compounds (Ezzariai et al. 2018). Bioremediation presents a promising strategy to remove harmful compounds from the contaminated environment and has been given attention as an efficient and cost-effective method compared to conventional techniques routinely applied in WWTPs (Koch et al. 2021). Bacteria-mediated recovery of chemicals using extremophiles is an environmentally friendly approach to clean antibiotics from contaminated sites (Morikawa 2006). Several studies have explored this approach with promising outcomes. For example, a study conducted by Al-Gheethi et al. suggests the use of *Bacillus subtilis* 1556WTNC for the successful removal of beta-lactams; cephalexin, ceftaroline, ampicillin, and amoxicillin from wastewater (Al-Gheethi et al. 2014). Algae has also been found to be safe and cost-effective in bioremediation of contaminants, including antibiotics (Tasho and Cho 2016). Antibiotics such as cefradine, cephalexin, cefixime, and ceftazidime can be efficiently removed by the green alga *Chlorella pyrenoidosa* (Yu et al. 2017). Recent reviews show that published studies done in the past few years have also demonstrated the prospects of using fungi in biodegradation of pharmaceuticals and pesticides (Naghdi et al. 2018).

## Future perspectives

There is a growing body of evidence demonstrating that the routine discharge of antibiotic compounds and their metabolites from a variety of sources has loaded multiple antimicrobials, including β-lactams, macrolides, quinolones, aminoglycosides, sulfonamides, trimethoprim, and tetracyclines residues into environmental matrices in many regions across the world. Despite numerous studies on the impact of their contamination, the individual and combined health effects of antibiotics on living organisms, including human beings, and a more comprehensive understanding of their potential risks in the environment are subject to further investigation.

Culture independent techniques are currently available for the detection of resistance genes or gene families, especially polymerase chain reaction (PCR) and/or quantitative polymerase chain reaction (qPCR)) and have contributed to the expansion of knowledge on the ARGs’ diversity, and abundance in aqueous environments as antimicrobial resistance reservoirs. In addition, intensifying the application of targeted functional and sequence-based metagenomics and new metagenomics-based studies are likely to reveal more insights into the occurrence and distribution of ARGs in wastewater.

To reduce the threat of the escalation of antimicrobial resistance facilitated by the aqueous environment, strict threshold limits for antibiotic release from point sources such as hospitals and animal husbandry, as well as thresholds for release of other pharmaceuticals, and biocides that drive co-selection of resistance, need to be established and applied universally. Further, diverse studies on the role of environmental antibiotic contamination and its correlation to the development of antibiotic resistance would aid in the formulation and strengthening of the intervention measures.

Given the threats posed by antimicrobial resistance to human health, it would be useful to intensify research on the prospective bioremediation approaches to remove environmental antibiotics to complement the variety of technologies that are applied to remove antibiotics and ARGs in wastewater treatment facilities.

Although a combination of approaches to remove antibiotics and ARGs from WWTPs has yielded promising outcomes, the adoption of novel therapeutic strategies, either alone or in conjunction with traditional therapies to control bacterial infections, offers a multifaceted approach to slow down the rapidly growing resistance to antimicrobial drugs in bacteria.

## Conclusions

The environment constituted by hospital effluents and wastewater treatment plants is rich in antimicrobial micro contaminants and creates selection pressure, leading to the emergence of antimicrobial resistance by providing an ideal platform for the residence and interaction of antibiotics, bacteria, and resistance genes. The absence of standardized regulations to monitor these microcontaminants has contributed to the escalation of antibiotic resistance in the environment. Evidence suggests that effluent from WWTPs is a reservoir of ARGs and is pivotal in their dissemination to both commensal and pathogenic bacteria in the receiving environments facilitated by horizontal gene transfer. The development of antimicrobial resistance spurred by antibiotics and other stressors in the environment raises concern due to the likelihood of simultaneous transmission of virulence and resistance determinants to multiple antibiotic classes by mobile genetic elements in bacteria, which may directly or indirectly reach human and animal hosts. The progression of AR in the environment presents a considerable challenge to the successful achievement of the One Health initiative envisaged by the World Health Organization as well as the full realization of the United Nations Sustainable Development Goals. Meanwhile, advanced technologies applied to eliminate antibiotics and ARGs from WWTPs are important to mitigate the adverse effects of such toxicants on aquatic environment.

## Data Availability

All relevant data are within the manuscript.

## References

[CR1] Al Aukidy M, Verlicchi P, Jelic A, Petrovic M, Barcelò D (2012). Monitoring release of pharmaceutical compounds: occurrence and environmental risk assessment of two WWTP effluents and their receiving bodies in the Po Valley, Italy. Sci Total Environ.

[CR2] Aldred KJ, Kerns RJ, Osheroff N (2014). Mechanism of quinolone action and resistance. Biochemistry.

[CR3] Alexander J, Bollmann A, Seitz W, Schwartz T (2015). Microbiological characterization of aquatic microbiomes targeting taxonomical marker genes and antibiotic resistance genes of opportunistic bacteria. Sci Total Environ.

[CR4] Al-Gheethi AA, Norli I, Lalung J, Azlan AM, Nur Farehah Z (2014). Biosorption of heavy metals and cephalexin from secondary effluents by tolerant bacteria. Clean Technol Environ Policy.

[CR5] Alvarino T, Nastold P, Suarez S, Omil F, Corvini P, Bouju H (2016). Role of biotransformation, sorption and mineralization of 14&nbsp;C-labelled sulfamethoxazole under different redox conditions. Sci Total Environ.

[CR6] Amador PP, Fernandes RM, Prudencio MC, Barreto MP, Duarte IM (2015). Antibiotic resistance in wastewater: occurrence and fate of Enterobacteriaceae producers of class A and class C beta-lactamases. J Environ Sci Health A.

[CR7] Ambler RP (1980). The structure of beta-lactamases. Philos Trans R Soc Lond B Biol Sci.

[CR8] Aminov RI, Garrigues-Jeanjean N, Mackie RI (2001). Molecular ecology of tetracycline resistance: development and validation of primers for detection of tetracycline resistance genes encoding ribosomal protection proteins. Appl Environ Microbiol.

[CR9] Aoki W, Ueda M (2013). Characterization of antimicrobial peptides toward the development of novel antibiotics. Pharmaceuticals (Basel).

[CR10] Araújo C, Torres C, Silva N, Carneiro C, Gonçalves A, Radhouani H, Correia S, da Costa PM, Paccheco R, Zarazaga M (2010). Vancomycin-resistant enterococci from Portuguese wastewater treatment plants. Basic Microbiol.

[CR11] Arlet G, Jacoby GA (2002). Plasmid-determined AmpC-type -lactamases. Antimicrob Agents Chemother.

[CR12] Auerbach EA, Seyfried EE, McMahon KD (2007). Tetracycline resistance genes in activated sludge wastewater treatment plants. Water Res.

[CR13] Azanu D, Styrishave B, Darko G, Weisser JJ, Abaidoo RC (2018). Occurrence and risk assessment of antibiotics in water and lettuce in Ghana. Sci Total Environ.

[CR14] Azanu D, Styrishave B, Darko G, Weisser JJ, Abaidoo RC (2018). Occurrence and risk assessment of antibiotics in water and lettuce in Ghana. Sci Total Environ.

[CR15] Baker SM, McLachlan JB, Morici LA (2020). Immunological considerations in the development of *Pseudomonas aeruginosa* vaccines. Hum Vaccin Immunother.

[CR16] Baquero F, Martinez JL, Canton R (2008). Antibiotics and antibiotic resistance in water environments. Curr Opin Biotechnol.

[CR17] Barancheshme F, Munir M (2018). Strategies to combat antibiotic resistance in the wastewater treatment plants. Front Microbiol.

[CR18] Barr V, Barr K, Millar MR, Lacey RW (1986). Beta-lactam antibiotics increase the frequency of plasmid transfer in *Staphylococcus aureus*. J Antimicrob Chemother.

[CR19] Bartlett JG (2005). 2005-6 Pocket book of infectious disease therapy.

[CR20] Bartrons M, Penuelas J (2017). Pharmaceuticals and personal-care products in plants. Trends Plant Sci.

[CR21] Batah R, Loucif L, Olaitan AO, Boutefnouchet N, Allag H, Rolain JM (2015). Outbreak of *Serratia marcescens* coproducing ArmA and CTX-M-15 mediated high levels of resistance to aminoglycoside and extended-spectrum beta-lactamases, Algeria. Microb Drug Resist.

[CR22] Bautitz IR, Nogueira RFP (2007). Degradation of tetracycline by photo-Fenton process—solar irradiation and matrix effects. Photochem Photobiol A.

[CR23] Belguesmia Y, Spano G, Drider D (2021). Potentiating effects of leaderless enterocin DD14 in combination with methicillin on clinical methicillin-resistant *Staphylococcus aureus* S1 strain. Microbiol Res.

[CR24] Bengtsson P, Kristiansson E, Larsson D (2018). Environmental factors influencing the development and spread of antibiotic resistance. FEMS Microbiol Rev.

[CR25] Bengtsson-Palme J, Hammaren R, Pal C, Östman M, Björlenius B, Flach C-F, Fick J, Kristiansson E, Tysklind M, Larsson DJ (2016). Elucidating selection processes for antibiotic resistance in sewage treatment plants using metagenomics. Sci Total Environ.

[CR26] Berendonk TU, Manaia CM, Merlin C, Fatta-Kassinos D, Cytryn E, Walsh F, Burgmann H, Sorum H, Norstrom M, Pons MN, Kreuzinger N, Huovinen P, Stefani S, Schwartz T, Kisand V, Baquero F, Martinez JL (2015). Tackling antibiotic resistance: the environmental framework. Nat Rev Microbiol.

[CR27] Berglund B (2015). Environmental dissemination of antibiotic resistance genes and correlation to anthropogenic contamination with antibiotics. Infect Ecol Epidemiol.

[CR28] Berglund B, Fick J, Lindgren PE (2015). Urban wastewater effluent increases antibiotic resistance gene concentrations in a receiving northern European river. Environ Toxicol Chem.

[CR29] Birošová L, Mackuľak T, Bodík I, Ryba J, Škubák J, Grabic R (2014). Pilot study of seasonal occurrence and distribution of antibiotics and drug resistant bacteria in wastewater treatment plants in Slovakia. Sci Total Environ.

[CR30] Biswal BK, Mazza A, Masson L, Gehr R, Frigon D (2014). Impact of wastewater treatment processes on antimicrobial resistance genes and their co-occurrence with virulence genes in *Escherichia coli*. Water Res.

[CR31] Blair JM, Webber MA, Baylay AJ, Ogbolu DO, Piddock LJ (2015). Molecular mechanisms of antibiotic resistance. Nat Rev Microbiol.

[CR32] Bönemann G, Stiens M, Pühler A, Schlüter A (2006). Mobilizable IncQ-related plasmid carrying a new quinolone resistance gene, qnrS2, isolated from the bacterial community of a wastewater treatment plant. Antimicrob Agents Chemother.

[CR33] Bouju H, Ricken B, Beffa T, Corvini PF, Kolvenbach BA (2012). Isolation of bacterial strains capable of sulfamethoxazole mineralization from an acclimated membrane bioreactor. Appl Environ Microbiol.

[CR34] Bradford PA (2001). Extended-spectrum β-lactamases in the 21st century: characterization, epidemiology, and detection of this important resistance threat. Clin Microbiol Rev.

[CR300] Brown AN, Smith K, Samuels TA, Lu J, Obare SO, Scott ME (2012) Nanoparticles functionalized with ampicillin destroy multiple antibiotic-resistant isolates of Pseudomonas aeruginosa and Enterobacter aerogenes and methicillin-resistant Staphylococcus aureus. Appl Environ Microbiol 78:2768–2774 10.1128/AEM.06513-11 PMC3318834 22286985

[CR35] Bunce J, Hellyer P (2018). Antibiotic resistance and antibiotic prescribing by dentists in England 2007–2016. Br Dent J.

[CR36] Bush K, Jacoby GA (2010). Updated functional classification of beta-lactamases. Antimicrob Agents Chemother.

[CR37] Cahill N, O’Connor L, Mahon B, Varley A, McGrath E, Ryan P, Cormican M, Brehony C, Jolley KA, Maiden MC, Brisse S, Morris D (2019). Hospital effluent: a reservoir for carbapenemase-producing Enterobacterales?. Sci Total Environ.

[CR38] Cao J, Hu Y, Liu F, Wang Y, Bi Y, Lv N, Li J, Zhu B, Gao GF (2020). Metagenomic analysis reveals the microbiome and resistome in migratory birds. Microbiome.

[CR39] Carraro E, Bonetta S, Bertino C, Lorenzi E, Bonetta S, Gilli G (2016). Hospital effluents management: chemical, physical, microbiological risks and legislation in different countries. J Environ Manage.

[CR40] Carvalho IT, Santos L (2016). Antibiotics in the aquatic environments: a review of the European scenario. Environ Int.

[CR41] Chatain-LY MH (2014). The factors affecting effectiveness of treatment in phages therapy. Front Microbiol.

[CR42] Chatterjee M, Anju CP, Biswas L, Anil Kumar V, Gopi Mohan C, Biswas R (2016). Antibiotic resistance in *Pseudomonas aeruginosa* and alternative therapeutic options. Int J Med Microbiol.

[CR43] Chen M, Ohman K, Metcalfe C, Ikonomou MG, Amatya PL, Wilson J (2006). Pharmaceuticals and endocrine disruptors in wastewater treatment effluents and in the water supply system of Calgary, Alberta, Canada. Water Qual Res J.

[CR44] Cheng D, Ngo HH, Guo W, Chang SW, Nguyen DD, Liu Y, Wei Q, Wei D (2020). A critical review on antibiotics and hormones in swine wastewater: water pollution problems and control approaches. J Hazard Mater.

[CR45] Chopra I, Roberts M (2001). Tetracycline antibiotics: mode of action, applications, molecular biology, and epidemiology of bacterial resistance. Microbiol Mol Biol Rev.

[CR46] Chowdhury MH, Diamond G, Ryan LK (2017) Synergy of antimicrobial peptides. In: Wang G (ed) Antimicrobial peptides: discovery, design novel therapeutic strategies. Nebraska, pp 188

[CR47] Chung PY, Khanum R (2017). Antimicrobial peptides as potential anti-biofilm agents against multidrug-resistant bacteria. Microbiol Immunol Infect Control.

[CR48] Citorik RJ, Mimee M, Lu TK (2014). Sequence-specific antimicrobials using efficiently delivered RNA-guided nucleases. Nat Biotechnol.

[CR49] Clokie MR, Millard AD, Letarov AV, Heaphy S (2011). Phages in nature. Bacteriophage.

[CR50] Collado N, Rodriguez-Mozaz S, Gros M, Rubirola A, Barcelo D, Comas J, Rodriguez-Roda I, Buttiglieri G (2014). Pharmaceuticals occurrence in a WWTP with significant industrial contribution and its input into the river system. Environ Pollut.

[CR51] Coque TM, Novais A, Carattoli A, Poirel L, Pitout J, Peixe L, Baquero F, Canton R, Nordmann P (2008). Dissemination of clonally related *Escherichia coli* strains expressing extended-spectrum beta-lactamase CTX-M-15. Emerg Infect Dis.

[CR52] Cornaglia G, Giamarellou H, Rossolini GM (2011). Metallo-β-lactamases: a last frontier for β-lactams?. Lancet Infect Dis.

[CR53] Cummings DE, Archer KF, Arriola DJ, Baker PA, Faucett KG, Laroya JB, Pfeil KL, Ryan CR, Ryan KR, Zuill DE (2011). Broad dissemination of plasmid-mediated quinolone resistance genes in sediments of two urban coastal wetlands. Environ Sci Technol.

[CR54] Cycoń M, Mrozik A, Piotrowska-Seget Z (2019). Antibiotics in the soil environment—degradation and their impact on microbial activity and diversity. Front Microbiol.

[CR55] Daghrir R, Drogui P (2013). Tetracycline antibiotics in the environment: a review. Environ Chem Lett.

[CR56] Dalkmann P, Broszat M, Siebe C, Willaschek E, Sakinc T, Huebner J, Amelung W, Grohmann E, Siemens J (2012). Accumulation of pharmaceuticals, *Enterococcus*, and resistance genes in soils irrigated with wastewater for zero to 100 years in central Mexico. PLoS ONE.

[CR57] Deng Y, Mao Y, Li B, Yang C, Zhang T (2016). Aerobic degradation of sulfadiazine by *Arthrobacter* spp.: kinetics, pathways, and genomic characterization. Environ Sci Technol.

[CR58] Deshpande AD, Baheti KG, Chatterjee NR (2004) Degradation of β-lactam antibiotics. Curr Sci 87(12):1684–1695

[CR59] Di Cesare A, Eckert EM, D’Urso S, Bertoni R, Gillan DC, Wattiez R, Corno G (2016). Co-occurrence of integrase 1, antibiotic and heavy metal resistance genes in municipal wastewater treatment plants. Water Res.

[CR60] Dolejska M, Villa L, Poirel L, Nordmann P, Carattoli A (2013). Complete sequencing of an IncHI1 plasmid encoding the carbapenemase NDM-1, the ArmA 16S RNA methylase and a resistance–nodulation–cell division/multidrug efflux pump. J Antimicrob Chemother.

[CR61] Dong H, Yuan X, Wang W, Qiang Z (2016). Occurrence and removal of antibiotics in ecological and conventional wastewater treatment processes: a field study. J Environ Manage.

[CR62] Döring G, Pier GB (2008). Vaccines and immunotherapy against *Pseudomonas aeruginosa*. Vaccine.

[CR63] Drlica K, Hiasa H, Kerns R, Malik M, Mustaev A, Zhao X (2009). Quinolones: action and resistance updated. Curr Top Med Chem.

[CR64] Du J, Geng J, Ren H, Ding L, Xu K, Zhang Y (2015). Variation of antibiotic resistance genes in municipal wastewater treatment plant with A2O-MBR system. Environ Sci Pollut Res.

[CR65] ECDC (2020) Annual report of the european antimicrobial resistance surveillance network antimicrobial resistance surveillance in Europe 2020. ECDC Stockholms

[CR66] Elsaesser A, Howard CV (2012). Toxicology of nanoparticles. Adv Drug Deliv Rev.

[CR67] Ezzariai A, Hafidi M, Khadra A, Aemig Q, El Fels L, Barret M, Merlina G, Patureau D, Pinelli E (2018). Human and veterinary antibiotics during composting of sludge or manure: global perspectives on persistence, degradation, and resistance genes. J Hazard Mater.

[CR68] Felis E, Kalka J, Sochacki A, Kowalska K, Bajkacz S, Harnisz M, Korzeniewska E (2020). Antimicrobial pharmaceuticals in the aquatic environment-occurrence and environmental implications. Eur J Pharmacol.

[CR69] Forcella C, Pellegrini C, Celenza G, Segatore B, Calabrese R, Tavio M, Amicosante G, Perilli M (2010). Qnr B9 in association with TEM-116 extended-spectrum beta-lactamase in *Citrobacter freundii* isolated from sewage effluent: first report from Italy. J Chemother.

[CR70] Gao P, Munir M, Xagoraraki I (2012). Correlation of tetracycline and sulfonamide antibiotics with corresponding resistance genes and resistant bacteria in a conventional municipal wastewater treatment plant. Sci Total Environ.

[CR71] Gbylik-Sikorska M, Posyniak A, Sniegocki T, Zmudzki J (2015). Liquid chromatography–tandem mass spectrometry multiclass method for the determination of antibiotics residues in water samples from water supply systems in food-producing animal farms. Chemosphere.

[CR72] Gelband H, Miller-Petrie M, Suraj P, Sumanth G, Levinson J, Barter D, White A, Laxminarayan R (2015) State of the world's antibiotics. Center for Disease Dynamics, Ecomomics and Policy. CDDEP, Washington D.C.

[CR73] Genereux DP, Bergstrom CT (2005) Evolution in action: understanding antibiotic resistance. In: Cracraft J, Bybee RW, editors. Evolutionary science and society: educating a new generation. AIBS, Washington DC, pp 145–153

[CR74] Göbel A, Thomsen A, McArdell CS, Joss A, Giger W (2005). Occurrence and sorption behavior of sulfonamides, macrolides, and trimethoprim in activated sludge treatment. Environ Sci Technol.

[CR75] Golovko O, Kumar V, Fedorova G, Randak T, Grabic R (2014). Seasonal changes in antibiotics, antidepressants/psychiatric drugs, antihistamines and lipid regulators in a wastewater treatment plant. Chemosphere.

[CR76] González-Alonso S, Merino LM, Esteban S, de Alda ML, Barceló D, Durán JJ, López-Martínez J, Aceña J, Pérez S, Mastroianni N (2017). Occurrence of pharmaceutical, recreational and psychotropic drug residues in surface water on the northern Antarctic Peninsula region. Environ Pollut.

[CR77] Gracia-Lor E, Sancho JV, Serrano R, Hernández F (2012). Occurrence and removal of pharmaceuticals in wastewater treatment plants at the Spanish Mediterranean area of Valencia. Chemosphere.

[CR78] Grassi L, Maisetta G, Esin S, Batoni G (2017). Combination strategies to enhance the efficacy of antimicrobial peptides against bacterial biofilms. Front Microbiol.

[CR79] Grave K, Torren-Edo J, Muller A, Greko C, Moulin G, Mackay D, Group E (2014). Variations in the sales and sales patterns of veterinary antimicrobial agents in 25 European countries. J Antimicrob Chemother.

[CR80] Gros M, Rodríguez-Mozaz S, Barceló D (2013). Rapid analysis of multiclass antibiotic residues and some of their metabolites in hospital, urban wastewater and river water by ultra-high-performance liquid chromatography coupled to quadrupole-linear ion trap tandem mass spectrometry. J Chromatogr A.

[CR81] Guerin E, Cambray G, Sanchez-Alberola N, Campoy S, Erill I, Da Re S, Gonzalez-Zorn B, Barbe J, Ploy MC, Mazel D (2009). The SOS response controls integron recombination. Science.

[CR82] Gullberg E, Cao S, Berg OG, Ilbäck C, Sandegren L, Hughes D, Andersson DI (2011). Selection of resistant bacteria at very low antibiotic concentrations. PLoS Pathog.

[CR83] Hancock RE, Haney EF, Gill EE (2016). The immunology of host defence peptides: beyond antimicrobial activity. Nat Rev Immunol.

[CR84] Hanna N, Sun P, Sun Q, Li X, Yang X, Ji X, Zou H, Ottoson J, Nilsson LE, Berglund B (2018). Presence of antibiotic residues in various environmental compartments of Shandong province in eastern China: its potential for resistance development and ecological and human risk. Environ Int.

[CR85] Harrabi M, Della Giustina SV, Aloulou F, Rodriguez-Mozaz S, Barceló D, Elleuch B (2018). Analysis of multiclass antibiotic residues in urban wastewater in Tunisia. Environ Nanatechnol Monit.

[CR86] He K, Blaney L (2015). Systematic optimization of an SPE with HPLC-FLD method for fluoroquinolone detection in wastewater. J Hazard Mater.

[CR87] Hei Y, Zhang H, Tan N, Zhou Y, Wei X, Hu C, Liu Y, Wang L, Qi J, Gao J-MJMR (2021). Antimicrobial activity and biosynthetic potential of cultivable actinomycetes associated with Lichen symbiosis from Qinghai-Tibet Plateau. Microbiol Res.

[CR88] Hendriksen RS, Munk P, Njage P, van Bunnik B, McNally L, Lukjancenko O, Roder T, Nieuwenhuijse D, Pedersen SK, Kjeldgaard J, Kaas RS, Clausen P, Vogt JK, Leekitcharoenphon P, van de Schans MGM, Zuidema T, de Roda Husman AM, Rasmussen S, Petersen B, Amid C, Cochrane G, Sicheritz-Ponten T, Schmitt H, Alvarez JRM, Aidara-Kane A, Pamp SJ, Lund O, Hald T, Woolhouse M, Koopmans MP, Vigre H, Petersen TN, Aarestrup FM, c. Global Sewage Surveillance project (2019). Global monitoring of antimicrobial resistance based on metagenomics analyses of urban sewage. Nat Commun.

[CR89] Henriques IS, Fonseca F, Alves A, Saavedra MJ, Correia A (2006). Occurrence and diversity of integrons and β-lactamase genes among ampicillin-resistant isolates from estuarine waters. Res Microbiol.

[CR90] Hirt H, Gorr S-U (2013). Antimicrobial peptide GL13K is effective in reducing biofilms of *Pseudomonas aeruginosa*. Antimicrob Agents Chemother.

[CR91] Hooper LV, Gordon JI (2001). Commensal host-bacterial relationships in the gut. Sci Total Environ.

[CR92] Hughes SR, Kay P, Brown LE (2013). Global synthesis and critical evaluation of pharmaceutical data sets collected from river systems. Environ Sci Technol.

[CR93] Jacoby GA (2005). Mechanisms of resistance to quinolones. Clin Infect Dis.

[CR94] Jacoby G (2009). AmpC B-lactamases clin. Microbiol Rev Jan.

[CR95] Jacoby GA, Munoz-Price LS (2005). The new beta-lactamases. N Engl J Med.

[CR96] Jacoby GA, Griffin CM, Hooper DC (2011). *Citrobacter* spp. as a source of qnrB alleles. Antimicrob Agents.

[CR97] Jeevanandam J, Barhoum A, Chan YS, Dufresne A, Danquah MK (2018). Review on nanoparticles and nanostructured materials: history, sources, toxicity and regulations. Beilstein J Nanotechnol.

[CR98] Jia Y, Khanal SK, Zhang H, Chen GH, Lu H (2017). Sulfamethoxazole degradation in anaerobic sulfate-reducing bacteria sludge system. Water Res.

[CR99] Jiang B, Li A, Cui D, Cai R, Ma F, Wang Y (2014). Biodegradation and metabolic pathway of sulfamethoxazole by *Pseudomonas psychrophila* HA-4, a newly isolated cold-adapted sulfamethoxazole-degrading bacterium. Appl Microbiol Biotechnol.

[CR100] Jiao YN, Zhou ZC, Chen T, Wei YY, Zheng J, Gao RX, Chen H (2018). Biomarkers of antibiotic resistance genes during seasonal changes in wastewater treatment systems. Environ Pollut.

[CR101] Jin JS, Kwon KT, Moon DC, Lee JC (2009). Emergence of 16S rRNA methylase rmtA in colistin-only-sensitive *Pseudomonas aeruginosa* in South Korea. Int J Antimicrob Agents.

[CR102] Johnson AP, Woodford N (2013). Global spread of antibiotic resistance: the example of New Delhi metallo-β-lactamase (NDM)-mediated carbapenem resistance. J Med Microbiol.

[CR103] Juricova H, Matiasovicova J, Kubasova T, Cejkova D, Rychlik I (2021). The distribution of antibiotic resistance genes in chicken gut microbiota commensals. Sci Rep.

[CR104] Karkman A, Pärnänen K, Larsson DJ (2019). Fecal pollution can explain antibiotic resistance gene abundances in anthropogenically impacted environments. Nat Commun.

[CR105] Kasprzyk-Hordern B, Dinsdale RM, Guwy AJ (2009). The removal of pharmaceuticals, personal care products, endocrine disruptors and illicit drugs during wastewater treatment and its impact on the quality of receiving waters. Water Res.

[CR106] Kassotaki E, Buttiglieri G, Ferrando-Climent L, Rodriguez-Roda I, Pijuan M (2016). Enhanced sulfamethoxazole degradation through ammonia oxidizing bacteria co-metabolism and fate of transformation products. Water Res.

[CR107] Kemper N (2008). Veterinary antibiotics in the aquatic and terrestrial environment. Ecol Ind.

[CR108] Khan FA, Soderquist B, Jass J (2019). Prevalence and diversity of antibiotic resistance genes in swedish aquatic environments impacted by household and hospital wastewater. Front Microbiol.

[CR109] Kim S, Jensen JN, Aga DS, Weber AS (2007). Tetracycline as a selector for resistant bacteria in activated sludge. Chemosphere.

[CR110] Kinney CA, Heuvel BV (2020). Translocation of pharmaceuticals and personal care products after land application of biosolids. Curr Opin Environ Sci Health Sci.

[CR111] Koch N, Islam NF, Sonowal S, Prasad R, Sarma H (2021). Environmental antibiotics and resistance genes as emerging contaminants: methods of detection and bioremediation. Curr Res Microb Sci.

[CR112] Korzeniewska E, Harnisz M (2013). Beta-lactamase-producing Enterobacteriaceae in hospital effluents. J Environ Manage.

[CR113] Korzeniewska E, Harnisz M (2018). Relationship between modification of activated sludge wastewater treatment and changes in antibiotic resistance of bacteria. Sci Total Environ.

[CR114] Korzeniewska E, Harnisz M (2020). Sources, occurrence, and environmental risk assessment of antibiotics and antimicrobial-resistant bacteria in aquatic environments of Poland. Polish river basins and lakes–part II.

[CR115] Kosma CI, Lambropoulou DA, Albanis TA (2014). Investigation of PPCPs in wastewater treatment plants in Greece: occurrence, removal and environmental risk assessment. Sci Total Environ.

[CR116] Kristiansson E, Fick J, Janzon A, Grabic R, Rutgersson C, Weijdegard B, Soderstrom H, Larsson DG (2011). Pyrosequencing of antibiotic-contaminated river sediments reveals high levels of resistance and gene transfer elements. PLoS ONE.

[CR117] Krzeminski P, Tomei MC, Karaolia P, Langenhoff A, Almeida CMR, Felis E, Gritten F, Andersen HR, Fernandes T, Manaia CM, Rizzo L, Fatta-Kassinos D (2019). Performance of secondary wastewater treatment methods for the removal of contaminants of emerging concern implicated in crop uptake and antibiotic resistance spread: a review. Sci Total Environ.

[CR118] Kumar A, Patyal A, Panda A (2018). Sub-therapeutic use of antibiotics in animal feed and their potential impact on environmental and human health: a comprehensive review. Anim Feed Sci Technol.

[CR119] Kumar M, Jaiswal S, Sodhi KK, Shree P, Singh DK, Agrawal PK, Shukla P (2019). Antibiotics bioremediation: perspectives on its ecotoxicity and resistance. Environ Int.

[CR120] Kümmerer K (2009). Antibiotics in the aquatic environment–a review–part I. Chemosphere.

[CR121] Labbate M, Case RJ, Stokes HW (2009). The integron/gene cassette system: an active player in bacterial adaptation. Methods Mol Biol.

[CR122] Lara-Martín PA, González-Mazo E, Petrovic M, Barceló D, Brownawell BJ (2014). Occurrence, distribution and partitioning of nonionic surfactants and pharmaceuticals in the urbanized Long Island Sound Estuary (NY). Mar Pollut Bull.

[CR123] Larcher S, Yargeau V (2011). Biodegradation of sulfamethoxazole by individual and mixed bacteria. Appl Microbiol Biotechnol.

[CR124] Larsson DG, de Pedro C, Paxeus N (2007). Effluent from drug manufactures contains extremely high levels of pharmaceuticals. J Hazard Mater.

[CR125] Leclercq R (2002). Mechanisms of resistance to macrolides and lincosamides: nature of the resistance elements and their clinical implications. Clin Infect Dis.

[CR126] Leng Y, Bao J, Chang G, Zheng H, Li X, Du J, Snow D, Li X (2016). Biotransformation of tetracycline by a novel bacterial strain *Stenotrophomonas maltophilia* DT1. J Hazard Mater.

[CR127] Leng Y, Bao J, Song D, Li J, Ye M, Li X (2017). Background nutrients affect the biotransformation of tetracycline by *Stenotrophomonas maltophilia* as revealed by genomics and proteomics. Environ Sci Technol.

[CR128] Levy SB (2002). The 2000 Garrod lecture. Factors impacting on the problem of antibiotic resistance. J Antimicrob Chemother.

[CR129] Li WC (2014). Occurrence, sources, and fate of pharmaceuticals in aquatic environment and soil. Environ Pollut.

[CR130] Li B, Zhang T (2011). Mass flows and removal of antibiotics in two municipal wastewater treatment plants. Chemosphere.

[CR131] Li D, Yang M, Hu J, Ren L, Zhang Y, Li K (2008). Determination and fate of oxytetracycline and related compounds in oxytetracycline production wastewater and the receiving river. Environ Toxicol Chem.

[CR132] Li D, Yu T, Zhang Y, Yang M, Li Z, Liu M, Qi R (2010). Antibiotic resistance characteristics of environmental bacteria from an oxytetracycline production wastewater treatment plant and the receiving river. Appl Environ Microbiol.

[CR133] Lin AY, Yu TH, Lateef SK (2009). Removal of pharmaceuticals in secondary wastewater treatment processes in Taiwan. J Hazard Mater.

[CR134] Lincopan N, Neves P, Mamizuka EM, Levy CE (2010). Balanoposthitis caused by *Pseudomonas aeruginosa* co-producing metallo-β-lactamase and 16S rRNA methylase in children with hematological malignancies. Int J Infect Dis.

[CR135] Lindberg R, Jarnheimer P-Ã, Olsen B, Johansson M, Tysklind M (2004). Determination of antibiotic substances in hospital sewage water using solid phase extraction and liquid chromatography/mass spectrometry and group analogue internal standards. Chemosphere.

[CR136] Lindberg RH, Olofsson U, Rendahl P, Johansson MI, Tysklind M, Andersson BA (2006). Behavior of fluoroquinolones and trimethoprim during mechanical, chemical, and active sludge treatment of sewage water and digestion of sludge. Environ Sci Technol.

[CR137] Lindberg RH, Bjorklund K, Rendahl P, Johansson MI, Tysklind M, Andersson BA (2007). Environmental risk assessment of antibiotics in the Swedish environment with emphasis on sewage treatment plants. Water Res.

[CR138] Liu J, Keelan P, Bennett PM, Enne VI (2009). Characterization of a novel macrolide efflux gene, mef (B), found linked to sul3 in porcine *Escherichia coli*. Antimicrob Chemother.

[CR139] Löffler D, Ternes TA (2003). Analytical method for the determination of the aminoglycoside gentamicin in hospital wastewater via liquid chromatography–electrospray-tandem mass spectrometry. Chromatogr A.

[CR140] Loos R, Carvalho R, Antonio DC, Comero S, Locoro G, Tavazzi S, Paracchini B, Ghiani M, Lettieri T, Blaha L, Jarosova B, Voorspoels S, Servaes K, Haglund P, Fick J, Lindberg RH, Schwesig D, Gawlik BM (2013). EU-wide monitoring survey on emerging polar organic contaminants in wastewater treatment plant effluents. Water Res.

[CR141] Loos R, Marinov D, Sanseverino I, Napierska D, Lettieri T (2018). Review of the 1st watch list under the water framework directive and recommendations for the 2nd watch list.

[CR142] Lorenzo P, Adriana A, Jessica S, Carles B, Marinella F, Marta L, Luis BJ, Pierre S (2018). Antibiotic resistance in urban and hospital wastewaters and their impact on a receiving freshwater ecosystem. Chemosphere.

[CR143] Majewsky M, Wagner D, Delay M, Bräse S, Yargeau V, Horn H (2014). Antibacterial activity of sulfamethoxazole transformation products (TPs): general relevance for sulfonamide TPs modified at the para position. Chem Res Toxicol.

[CR144] Managaki S, Murata A, Takada H, Tuyen BC, Chiem NH (2007). Distribution of macrolides, sulfonamides, and trimethoprim in tropical waters: ubiquitous occurrence of veterinary antibiotics in the Mekong Delta. Environ Sci Technol.

[CR145] Mao F, Liu X, Wu K, Zhou C, Si Y (2018). Biodegradation of sulfonamides by *Shewanella oneidensis* MR-1 and *Shewanella* sp. strain MR-4. Biodegradation.

[CR146] Markley J, Wencewicz T (2018). Tetracycline-inactivating enzymes. Front Microbiol.

[CR147] Marti E, Jofre J, Balcazar JL (2013). Prevalence of antibiotic resistance genes and bacterial community composition in a river influenced by a wastewater treatment plant. PLoS ONE.

[CR148] Matongo S, Birungi G, Moodley B, Ndungu P (2015). Occurrence of selected pharmaceuticals in water and sediment of Umgeni River, KwaZulu-Natal, South Africa. Environ Sci Pollut Res Int.

[CR149] Mazel D (2006). Integrons: agents of bacterial evolution. Nat Rev Microbiol.

[CR150] McArdell CS, Molnar E, Suter MJ, Giger W (2003). Occurrence and fate of macrolide antibiotics in wastewater treatment plants and in the Glatt Valley watershed, Switzerland. Environ Sci Technol.

[CR151] Mendoza A, Aceña J, Pérez S, De Alda ML, Barceló D, Gil A, Valcárcel Y (2015). Pharmaceuticals and iodinated contrast media in a hospital wastewater: a case study to analyse their presence and characterise their environmental risk and hazard. Environ Res.

[CR152] Michael I, Rizzo L, McArdell C, Manaia C, Merlin C, Schwartz T, Dagot C, Fatta-Kassinos D (2013). Urban wastewater treatment plants as hotspots for the release of antibiotics in the environment: a review. Water Res.

[CR153] Minh TB, Leung HW, Loi IH, Chan WH, So MK, Mao JQ, Choi D, Lam JC, Zheng G, Martin M, Lee JH, Lam PK, Richardson BJ (2009). Antibiotics in the Hong Kong metropolitan area: ubiquitous distribution and fate in Victoria Harbour. Mar Pollut Bull.

[CR154] Mohatt JL, Hu L, Finneran KT, Strathmann TJ (2011). Microbially mediated abiotic transformation of the antimicrobial agent sulfamethoxazole under iron-reducing soil conditions. Environ Sci Technol.

[CR155] Moreno-González R, Rodríguez-Mozaz S, Gros M, Pérez-Cánovas E, Barceló D, León VM (2014). Input of pharmaceuticals through coastal surface watercourses into a Mediterranean lagoon (Mar Menor, SE Spain): sources and seasonal variations. Sci Total Environ.

[CR156] Morikawa M (2006). Beneficial biofilm formation by industrial bacteria *Bacillus subtilis* and related species. J Biosci Bioeng.

[CR157] Morris J, Sulakvelidze A, Alavidze Z (2001). Bacteriophage therapy. Antimicrob Agents Chemother.

[CR158] Moura A, Henriques I, Ribeiro R, Correia A (2007). Prevalence and characterization of integrons from bacteria isolated from a slaughterhouse wastewater treatment plant. J Antimicrob Chemother.

[CR159] Munita JM, Arias CA (2016). Mechanisms of antibiotic resistance. Microbiol Spectr.

[CR160] Muramatsu H, Horii T, Takeshita A, Hashimoto H, Maekawa M (2005). Characterization of fluoroquinolone and carbapenem susceptibilities in clinical isolates of levofloxacin-resistant *Pseudomonas aeruginosa*. Chemotherapy.

[CR161] Naghdi M, Taheran M, Brar SK, Kermanshahi-Pour A, Verma M, Surampalli RY (2018). Removal of pharmaceutical compounds in water and wastewater using fungal oxidoreductase enzymes. Environ Pollut.

[CR162] Neudorf KD, Huang YN, Ragush CM, Yost CK, Jamieson RC, Truelstrup Hansen L (2017). Antibiotic resistance genes in municipal wastewater treatment systems and receiving waters in Arctic Canada. Sci Total Environ.

[CR163] Ngumba E, Gachanja A, Tuhkanen T (2016). Occurrence of selected antibiotics and antiretroviral drugs in Nairobi River Basin, Kenya. Sci Total Environ.

[CR164] Nguyen LN, Nghiem LD, Oh S (2018). Aerobic biotransformation of the antibiotic ciprofloxacin by *Bradyrhizobium* sp. isolated from activated sludge. Chemosphere.

[CR165] Nnadozie C, Kumari S, Bux F (2017). Status of pathogens, antibiotic resistance genes and antibiotic residues in wastewater treatment systems. Rev Environ Sci Bio/Technol.

[CR166] Oberoi AS, Jia Y, Zhang H, Khanal SK, Lu H (2019). Insights into the fate and removal of antibiotics in engineered biological treatment systems: a critical review. Environ Sci Technol.

[CR167] Opriş O, Soran M-L, Coman V, Copaciu F, Ristoiu D (2013). Determination of some frequently used antibiotics in waste waters using solid phase extraction followed by high performance liquid chromatography with diode array and mass spectrometry detection. Cent Eur J Chem.

[CR168] Örmälä A-M, Jalasvuori M (2013). Phage therapy: should bacterial resistance to phages be a concern. even in the long run? Bacteriophage.

[CR169] Osorio V, Marcé R, Pérez S, Ginebreda A, Cortina JL, Barceló D (2012). Occurrence and modeling of pharmaceuticals on a sewage-impacted Mediterranean river and their dynamics under different hydrological conditions. Sci Total Environ.

[CR170] Östman M, Lindberg RH, Fick J, Björn E, Tysklind M (2017). Screening of biocides, metals and antibiotics in Swedish sewage sludge and wastewater. Water Res.

[CR171] Papageorgiou M, Kosma C, Lambropoulou D (2016). Seasonal occurrence, removal, mass loading and environmental risk assessment of 55 pharmaceuticals and personal care products in a municipal wastewater treatment plant in Central Greece. Sci Total Environ.

[CR172] Park SC, Park Y, Hahm KS (2011). The role of antimicrobial peptides in preventing multidrug-resistant bacterial infections and biofilm formation. Int J Mol Sci.

[CR173] Pärnänen KM, Narciso-da-Rocha C, Kneis D, Berendonk TU, Cacace D, Do TT, Elpers C, Fatta-Kassinos D, Henriques I, Jaeger. TJSA  (2019). Antibiotic resistance in European wastewater treatment plants mirrors the pattern of clinical antibiotic resistance prevalence. J Sci Adv.

[CR174] Partridge SR (2015). Resistance mechanisms in Enterobacteriaceae. Pathology.

[CR175] Partridge SR, Tsafnat G, Coiera E, Iredell JR (2009). Gene cassettes and cassette arrays in mobile resistance integrons. FEMS Microbiol Rev.

[CR176] Pazda M, Kumirska J, Stepnowski P, Mulkiewicz E (2019). Antibiotic resistance genes identified in wastewater treatment plant systems—a review. Sci Total Environ.

[CR177] Pei R, Kim SC, Carlson KH, Pruden A (2006). Effect of river landscape on the sediment concentrations of antibiotics and corresponding antibiotic resistance genes (ARG). Water Res.

[CR178] Petrovic M, Gros M, Barcelo D (2006). Multi-residue analysis of pharmaceuticals in wastewater by ultra-performance liquid chromatography–quadrupole–time-of-flight mass spectrometry. Chromatogr A.

[CR179] Pires DP, Cleto S, Sillankorva S, Azeredo J, Lu TK (2016). Genetically Engineered phages: a review of advances over the last decade. Microbiol Mol Biol Rev.

[CR180] Poirel L, Rodriguez-Martinez JM, Mammeri H, Liard A, Nordmann P (2005). Origin of plasmid-mediated quinolone resistance determinant QnrA. Antimicrob Agents Chemother.

[CR181] Poirel L, Potron A, Nordmann P (2012). OXA-48-like carbapenemases: the phantom menace. J Antimicrob Chemother.

[CR182] Poirel L, Madec JY, Lupo A, Schink AK, Kieffer N, Nordmann P, Schwarz S (2018). Antimicrobial resistance in *Escherichia coli*. Microbiol Spectr.

[CR183] Prescott JF (2013). Sulfonamides, diaminopyrimidines, and their combinations. Antimicrob Therapy Vet Med.

[CR184] Qiu Q, Wang J, Yan Y, Roy B, Chen Y, Shang X, Dou T, Han L (2020). Metagenomic analysis reveals the distribution of antibiotic resistance genes in a large-scale population of healthy individuals and patients with varied diseases. Front Mol Biosci.

[CR185] Ramirez MS, Tolmasky ME (2010). Aminoglycoside modifying enzymes. Drug Resist Updates.

[CR186] Recchia GD, Hall RM (1995). Gene cassettes: a new class of mobile element. Microbiol (Reading).

[CR187] Reis PJ, Reis AC, Ricken B, Kolvenbach BA, Manaia CM, Corvini PF, Nunes OC (2014). Biodegradation of sulfamethoxazole and other sulfonamides by Achromobacter denitrificans PR1. J Hazard Mater.

[CR188] Reis PJM, Homem V, Alves A, Vilar VJP, Manaia CM, Nunes OC (2018). Insights on sulfamethoxazole bio-transformation by environmental Proteobacteria isolates. J Hazard Mater.

[CR189] Reis EO, Foureaux AFS, Rodrigues JS, Moreira VR, Lebron YA, Santos LV, Amaral MC, Lange LC (2019). Occurrence, removal and seasonal variation of pharmaceuticals in Brasilian drinking water treatment plants. Environ Pollut.

[CR190] Rejiba S, Aubry A, Petitfrere S, Jarlier V, Cambau E (2008). Contribution of ParE mutation and efflux to ciprofloxacin resistance in *Pseudomonas aeruginosa* clinical isolates. J Chemother.

[CR191] Riaz L, Mahmood T, Khalid A, Rashid A, Siddique MBA, Kamal A, Coyne MS (2018). Fluoroquinolones (FQs) in the environment: a review on their abundance, sorption and toxicity in soil. Chemosphere.

[CR192] Ribeiro AR, Sures B, Schmidt TC (2018). Cephalosporin antibiotics in the aquatic environment: a critical review of occurrence, fate, ecotoxicity and removal technologies. Environ Pollut.

[CR193] Ricci M, Lava R, Koleva B (2016). Matrix certified reference materials for environmental monitoring under the EU water framework directive: an update. Trends Anal Chem.

[CR194] Ricken B, Corvini PF, Cichocka D, Parisi M, Lenz M, Wyss D, Martinez-Lavanchy PM, Muller JA, Shahgaldian P, Tulli LG, Kohler HP, Kolvenbach BA (2013). Ipso-hydroxylation and subsequent fragmentation: a novel microbial strategy to eliminate sulfonamide antibiotics. Appl Environ Microbiol.

[CR195] Ricken B, Fellmann O, Kohler H-PE, Schaeffer A, Corvini PF-X, Kolvenbach BA (2015). Degradation of sulfonamide antibiotics by *Microbacterium* sp. strain BR1–elucidating the downstream pathway. New Biotechnol.

[CR196] Ricken B, Kolvenbach BA, Bergesch C, Benndorf D, Kroll K, Strnad H, Vlcek C, Adaixo R, Hammes F, Shahgaldian P, Schaffer A, Kohler HE, Corvini PF (2017). FMNH2-dependent monooxygenases initiate catabolism of sulfonamides in *Microbacterium* sp. strain BR1 subsisting on sulfonamide antibiotics. Sci Rep.

[CR197] Roberts MC (2005). Update on acquired tetracycline resistance genes. FEMS Microbiol Lett.

[CR198] Roberts MC (2008). Update on macrolide–lincosamide–streptogramin, ketolide, and oxazolidinone resistance genes. FEMS Microbiol Lett.

[CR199] Roberts AP, Mullany P (2009). A modular master on the move: the Tn916 family of mobile genetic elements. Trends Microbiol.

[CR200] Robicsek A, Jacoby GA, Hooper DC (2006). The worldwide emergence of plasmid-mediated quinolone resistance. Lancet Infect Dis.

[CR201] Robinson LJ, Verrett JN, Sorout N, Stavrinides J (2020). A broad-spectrum antibacterial natural product from the cystic fibrosis isolate, *Pantoea agglomeran*s Tx10. Microbiol Res.

[CR202] Rodriguez-Mozaz S, Vaz-Moreira I, Della Giustina SV, Llorca M, Barceló D, Schubert S, Berendonk TU, Michael-Kordatou I, Fatta-Kassinos D, Martinez JL (2020). Antibiotic residues in final effluents of European wastewater treatment plants and their impact on the aquatic environment. Environ Int.

[CR203] Rossmann J, Schubert S, Gurke R, Oertel R, Kirch W (2014). Simultaneous determination of most prescribed antibiotics in multiple urban wastewater by SPE-LC–MS/MS. J Chromatogr B.

[CR204] Ruff M, Mueller MS, Loos M, Singer HP (2015). Quantitative target and systematic non-target analysis of polar organic micro-pollutants along the river Rhine using high-resolution mass-spectrometry–identification of unknown sources and compounds. Water Res.

[CR205] Sabino YNV, Santana MF, Oyama LB, Santos FG, Moreira AJS, Huws SA, Mantovani HC (2019). Characterization of antibiotic resistance genes in the species of the rumen microbiota. Nat Commun.

[CR206] Sabri N, Schmitt H, Van der Zaan B, Gerritsen H, Zuidema T, Rijnaarts H, Langenhoff A (2020). Prevalence of antibiotics and antibiotic resistance genes in a wastewater effluent-receiving river in The Netherlands. Environ Chem Eng.

[CR207] Salomoni R, Leo P, Montemor AF, Rinaldi BG, Rodrigues M (2017). Antibacterial effect of silver nanoparticles in *Pseudomonas aeruginosa*. Nanotechnol Sci Appl.

[CR208] Santos LH, Gros M, Rodriguez-Mozaz S, Delerue-Matos C, Pena A, Barcelo D, Montenegro MC (2013). Contribution of hospital effluents to the load of pharmaceuticals in urban wastewaters: identification of ecologically relevant pharmaceuticals. Sci Total Environ.

[CR209] Schluter A, Szczepanowski R, Puhler A, Top EM (2007). Genomics of IncP-1 antibiotic resistance plasmids isolated from wastewater treatment plants provides evidence for a widely accessible drug resistance gene pool. FEMS Microbiol Rev.

[CR210] Segura PA, Francois M, Gagnon C, Sauve S (2009). Review of the occurrence of anti-infectives in contaminated wastewaters and natural and drinking waters. Environ Health Perspect.

[CR211] Seifrtova M, Novakova L, Lino C, Pena A, Solich P (2009). An overview of analytical methodologies for the determination of antibiotics in environmental waters. Anal Chim Acta.

[CR212] Sharma VK, Johnson N, Cizmas L, McDonald TJ, Kim H (2016). A review of the influence of treatment strategies on antibiotic resistant bacteria and antibiotic resistance genes. Chemosphere.

[CR213] Siqueira AK, Michael GB, Domingos DF, Ferraz MM, Ribeiro MG, Schwarz S, Leite DS (2016). Diversity of class 1 and 2 integrons detected in *Escherichia coli* isolates from diseased and apparently healthy dogs. Vet Microbiol.

[CR214] Spongberg AL, Witter JD (2008). Pharmaceutical compounds in the wastewater process stream in Northwest Ohio. Sci Total Environ.

[CR215] Stern A, Sorek R (2011). The phage-host arms race: shaping the evolution of microbes. BioEssays.

[CR216] Stoll C, Sidhu JP, Tiehm A, Toze S (2012). Prevalence of clinically relevant antibiotic resistance genes in surface water samples collected from Germany and Australia. Environ Sci Technol.

[CR217] Strahilevitz J, Jacoby GA, Hooper DC, Robicsek A (2009). Plasmid-mediated quinolone resistance: a multifaceted threat. Clin Microbiol Rev.

[CR218] Sunde M, Solheim H, Slettemeas JS (2008). Genetic linkage between class 1 integrons with the dfrA12-orfF-aadA2 cassette array and sul3 in *Escherichia coli*. Vet Microbiol.

[CR219] Suzuki S, Ogo M, Miller TW, Shimizu A, Takada H, Siringan MA (2013). Who possesses drug resistance genes in the aquatic environment?: sulfamethoxazole (SMX) resistance genes among the bacterial community in water environment of Metro-Manila, Philippines. Front Microbiol.

[CR220] Szczepanowski R, Linke B, Krahn I, Gartemann KH, Gutzkow T, Eichler W, Puhler A, Schluter A (2009). Detection of 140 clinically relevant antibiotic-resistance genes in the plasmid metagenome of wastewater treatment plant bacteria showing reduced susceptibility to selected antibiotics. Microbiol (Reading).

[CR221] Szymańska U, Wiergowski M, Sołtyszewski I, Kuzemko J, Wiergowska G, Woźniak MK (2019). Presence of antibiotics in the aquatic environment in Europe and their analytical monitoring: recent trends and perspectives. Microchem J.

[CR222] Tahrani L, Van Loco J, Ben Mansour H, Reyns T (2016). Occurrence of antibiotics in pharmaceutical industrial wastewater, wastewater treatment plant and sea waters in Tunisia. J Water Health Sci.

[CR223] Tam VH, Chang KT, Abdelraouf K, Brioso CG, Ameka M, McCaskey LA, Weston JS, Caeiro JP, Garey KW (2010). Prevalence, resistance mechanisms, and susceptibility of multidrug-resistant bloodstream isolates of *Pseudomonas aeruginosa*. Antimicrob Agents Chemother.

[CR224] Tang SS, Apisarnthanarak A, Hsu LY (2014). Mechanisms of beta-lactam antimicrobial resistance and epidemiology of major community- and healthcare-associated multidrug-resistant bacteria. Adv Drug Deliv Rev.

[CR225] Tasho RP, Cho JY (2016). Veterinary antibiotics in animal waste, its distribution in soil and uptake by plants: a review. Sci Total Environ.

[CR226] Tennstedt T, Szczepanowski R, Braun S, Puhler A, Schluter A (2003). Occurrence of integron-associated resistance gene cassettes located on antibiotic resistance plasmids isolated from a wastewater treatment plant. FEMS Microbiol Ecol.

[CR227] Ternes TA, Bonerz M, Herrmann N, Teiser B, Andersen HR (2007). Irrigation of treated wastewater in Braunschweig, Germany: an option to remove pharmaceuticals and musk fragrances. Chemosphere.

[CR228] Thai PK, Ky LX, Binh VN, Nhung PH, Nhan PT, Hieu NQ, Dang NTT, Tam NKB, Anh NTK (2018). Occurrence of antibiotic residues and antibiotic-resistant bacteria in effluents of pharmaceutical manufacturers and other sources around Hanoi. Vietnam Sci Total Environ.

[CR229] Toke O (2005). Antimicrobial peptides: new candidates in the fight against bacterial infections. Biopolymers.

[CR230] Touchon M, Moura de Sousa JA, Rocha EP (2017). Embracing the enemy: the diversification of microbial gene repertoires by phage-mediated horizontal gene transfer. Curr Opin Microbiol.

[CR231] Tuckman M, Petersen PJ, Howe AY, Orlowski M, Mullen S, Chan K, Bradford PA, Jones CH (2007). Occurrence of tetracycline resistance genes among Escherichia coli isolates from the phase 3 clinical trials for tigecycline. Antimicrob Agents Chemother.

[CR232] Tylová T, Flieger M, Olšovská J (2013). Determination of antibiotics in influents and effluents of wastewater-treatment-plants in the Czech Republic–development and application of the SPE and a UHPLC-ToFMS method. Anal Methods.

[CR233] Úbeda C, Maiques E, Knecht E, Lasa Í, Novick RP, Penadés JR (2005). Antibiotic-induced SOS response promotes horizontal dissemination of pathogenicity island‐encoded virulence factors in *staphylococci*. Mol Microbiol.

[CR234] Uluseker C, Kaster KM, Thorsen K, Basiry D, Shobana S, Jain M, Kumar G, Kommedal R, Pala-Ozkok I (2021). A review on occurrence and spread of antibiotic resistance in wastewaters and in wastewater treatment plants: mechanisms and perspectives. Front Microbiol.

[CR235] Valcarcel Y, Alonso SG, Rodriguez-Gil JL, Gil A, Catala M (2011). Detection of pharmaceutically active compounds in the rivers and tap water of the Madrid Region (Spain) and potential ecotoxicological risk. Chemosphere.

[CR236] Van TTH, Yidana Z, Smooker PM, Coloe PJ (2020). Antibiotic use in food animals worldwide, with a focus on Africa: pluses and minuses. Glob Antimicrob Resist.

[CR237] Vergeynst L, Haeck A, De Wispelaere P, Van Langenhove H, Demeestere K (2015). Multi-residue analysis of pharmaceuticals in wastewater by liquid chromatography–magnetic sector mass spectrometry: method quality assessment and application in a Belgian case study. Chemosphere.

[CR238] Verlicchi P, Al Aukidy M, Galletti A, Petrovic M, Barcelo D (2012). Hospital effluent: investigation of the concentrations and distribution of pharmaceuticals and environmental risk assessment. Sci Total Environ.

[CR239] Von Wintersdorff CJ, Penders J, Van Niekerk JM, Mills ND, Majumder S, Van Alphen LB, Savelkoul PH, Wolffs PF (2016). Dissemination of antimicrobial resistance in microbial ecosystems through horizontal gene transfer. Front Microbiol.

[CR240] Wachino J-i, Arakawa Y (2012). Exogenously acquired 16S rRNA methyltransferases found in aminoglycoside-resistant pathogenic Gram-negative bacteria: an update. Drug Resist Updates.

[CR241] Wang J, Wang S (2016). Removal of pharmaceuticals and personal care products (PPCPs) from wastewater: a review. J Environ Manage.

[CR242] Wang J, Wang S (2018). Microbial degradation of sulfamethoxazole in the environment. Appl Microbiol Biotechnol.

[CR243] Wang L, Hu C, Shao L (2017). The antimicrobial activity of nanoparticles: present situation and prospects for the future. Int J Nanomed.

[CR244] Wang Q, Wang P, Yang Q (2018). Occurrence and diversity of antibiotic resistance in untreated hospital wastewater. Sci Total Environ.

[CR245] Wang J, Chu L, Wojnárovits L, Takács E (2020). Occurrence and fate of antibiotics, antibiotic resistant genes (ARGs) and antibiotic resistant bacteria (ARB) in municipal wastewater treatment plant: an overview. Sci Total Environ.

[CR246] Watkinson A, Murby E, Costanzo S (2007). Removal of antibiotics in conventional and advanced wastewater treatment: implications for environmental discharge and wastewater recycling. Water Res.

[CR247] Watkinson AJ, Murby EJ, Kolpin DW, Costanzo SD (2009). The occurrence of antibiotics in an urban watershed: from wastewater to drinking water. Sci Total Environ.

[CR248] WHO (2020) Global antimicrobial resistance surveillance system (GLASS) report: early implementation 2020

[CR249] Xu J, Xu Y, Wang H, Guo C, Qiu H, He Y, Zhang Y, Li X, Meng W (2015). Occurrence of antibiotics and antibiotic resistance genes in a sewage treatment plant and its effluent-receiving river. Chemosphere.

[CR250] Xu YB, Hou MY, Li YF, Huang L, Ruan JJ, Zheng L, Qiao QX, Du QP (2017). Distribution of tetracycline resistance genes and AmpC beta-lactamase genes in representative non-urban sewage plants and correlations with treatment processes and heavy metals. Chemosphere.

[CR251] Yan Y, Li H, Fayyaz A, Gai Y (2022). Metagenomic and network analysis revealed wide distribution of antibiotic resistance genes in monkey gut microbiota. Microbiol Res.

[CR252] Yang S, Carlson K (2004). Solid-phase extraction–high-performance liquid chromatography–ion trap mass spectrometry for analysis of trace concentrations of macrolide antibiotics in natural and waste water matrices. Chromatogr A.

[CR253] Yang S, Cha J, Carlson K (2005). Simultaneous extraction and analysis of 11 tetracycline and sulfonamide antibiotics in influent and effluent domestic wastewater by solid-phase extraction and liquid chromatography-electrospray ionization tandem mass spectrometry. J Chromatogr A.

[CR254] Yang Y, Li B, Ju F, Zhang T (2013). Exploring variation of antibiotic resistance genes in activated sludge over a four-year period through a metagenomic approach. Environ Sci Technol.

[CR255] Yang Y, Li B, Zou S, Fang HH, Zhang T (2014). Fate of antibiotic resistance genes in sewage treatment plant revealed by metagenomic approach. Water Res.

[CR256] Yang F, Gu J, Yang L, Gao C, Jing H, Wang Y, Zeng H, Zou Q, Lv F, Zhang J (2017). Protective efficacy of the trivalent *Pseudomonas aeruginosa* vaccine candidate PcrV-OprI-Hcp1 in murine pneumonia and burn models. Sci Rep.

[CR257] Yildirimer L, Thanh NT, Loizidou M, Seifalian AM (2011). Toxicology and clinical potential of nanoparticles. Nano Today.

[CR258] Yu Y-s, Zhou H, Yang Q, Chen Y-g, Li L-j (2007). Widespread occurrence of aminoglycoside resistance due to ArmA methylase in imipenem-resistant *Acinetobacter baumannii* isolates in China. Antimicrob Chemother.

[CR259] Yu Y, Zhou Y, Wang Z, Torres OL, Guo R, Chen J (2017). Investigation of the removal mechanism of antibiotic ceftazidime by green algae and subsequent microbic impact assessment. Sci Rep.

[CR260] Zavascki AP, Carvalhaes CG, Picao RC, Gales AC (2010). Multidrug-resistant *Pseudomonas aeruginosa* and *Acinetobacter baumannii*: resistance mechanisms and implications for therapy. Expert Rev Anti Infect Ther.

[CR261] Zhang T, Li B (2011). Occurrence, transformation, and fate of antibiotics in municipal wastewater treatment plants. Crit Rev Environ Sci Technol.

[CR262] Zhang XX, Zhang T (2011). Occurrence, abundance, and diversity of tetracycline resistance genes in 15 sewage treatment plants across China and other global locations. Environ Sci Technol.

[CR263] Zhang XX, Zhang T, Zhang M, Fang HH, Cheng SP (2009). Characterization and quantification of class 1 integrons and associated gene cassettes in sewage treatment plants. Appl Microbiol Biotechnol.

[CR264] Zhang Y, Marrs CF, Simon C, Xi C (2009). Wastewater treatment contributes to selective increase of antibiotic resistance among *Acinetobacter* spp. Sci Total Environ.

[CR265] Zhang YB, Zhou J, Xu QM, Cheng JS, Luo YL, Yuan YJ (2016). Exogenous cofactors for the improvement of bioremoval and biotransformation of sulfamethoxazole by *Alcaligenes faecalis*. Sci Total Environ.

[CR266] Zhen X, Lundborg CS, Sun X, Hu X, Dong H (2019). Economic burden of antibiotic resistance in ESKAPE organisms: a systematic review. Antimicrob Resist Infect Control.

[CR267] Zhou Y, Yu H, Guo Q, Xu X, Ye X, Wu S, Guo Y, Wang M (2010). Distribution of 16S rRNA methylases among different species of Gram-negative bacilli with high-level resistance to aminoglycosides. Eur J Clin Microbiol Infect Dis.

[CR268] Zhou Z-C, Feng W-Q, Han Y, Zheng J, Chen T, Wei Y-Y, Gillings M, Zhu Y-G, Chen H (2018). Prevalence and transmission of antibiotic resistance and microbiota between humans and water environments. Environ Int.

[CR269] Zhou CS, Wu JW, Dong LL, Liu BF, Xing DF, Yang SS, Wu XK, Wang Q, Fan JN, Feng LP, Cao GL (2020). Removal of antibiotic resistant bacteria and antibiotic resistance genes in wastewater effluent by UV-activated persulfate. J Hazard Mater.

[CR270] Zhu YG, Johnson TA, Su JQ, Qiao M, Guo GX, Stedtfeld RD, Hashsham SA, Tiedje JM (2013). Diverse and abundant antibiotic resistance genes in Chinese swine farms. Proc Natl Acad Sci USA.

[CR271] Zhu T-t, Su Z-x, Lai W-x, Zhang Y-b (2021). and Y.-W. Liu. Insights into the fate and removal of antibiotics and antibiotic resistance genes using biological wastewater treatment technology. Sci Total Environ.

[CR272] Zurfluh K, Bagutti C, Brodmann P, Alt M, Schulze J, Fanning S, Stephan R, Nüesch-Inderbinen M (2017). Wastewater is a reservoir for clinically relevant carbapenemase-and 16s rRNA methylase-producing Enterobacteriaceae. Int J Antimicrob Agents.

